# Postnatal Smad3 Inactivation in Murine Smooth Muscle Cells Elicits a Temporally and Regionally Distinct Transcriptional Response

**DOI:** 10.3389/fcvm.2022.826495

**Published:** 2022-04-08

**Authors:** Emily E. Bramel, Tyler J. Creamer, Muzna Saqib, Wendy A. Camejo Nunez, Rustam Bagirzadeh, LaToya Ann Roker, Loyal A. Goff, Elena Gallo MacFarlane

**Affiliations:** ^1^McKusick-Nathans Department of Genetic Medicine, Johns Hopkins University School of Medicine, Baltimore, MD, United States; ^2^Predoctoral Training in Human Genetics and Molecular Biology, Johns Hopkins University School of Medicine, Baltimore, MD, United States; ^3^School of Medicine Microscope Facility, Johns Hopkins University School of Medicine, Baltimore, MD, United States; ^4^Solomon H. Snyder Department of Neuroscience, Johns Hopkins University School of Medicine, Baltimore, MD, United States; ^5^Kavli Neuroscience Discovery Institute, Johns Hopkins University School of Medicine, Baltimore, MD, United States; ^6^Department of Surgery, Johns Hopkins University School of Medicine, Baltimore, MD, United States

**Keywords:** TGF-β, Smad3, scRNA seq, Loeys-Dietz syndrome, aortic aneurysm, smooth muscle

## Abstract

Heterozygous, loss of function mutations in positive regulators of the Transforming Growth Factor-β (TGF-β) pathway cause hereditary forms of thoracic aortic aneurysm. It is unclear whether and how the initial signaling deficiency triggers secondary signaling upregulation in the remaining functional branches of the pathway, and if this contributes to maladaptive vascular remodeling. To examine this process in a mouse model in which time-controlled, partial interference with postnatal TGF-β signaling in vascular smooth muscle cells (VSMCs) could be assessed, we used a VSMC-specific tamoxifen-inducible system, and a conditional allele, to inactivate *Smad3* at 6 weeks of age, after completion of perinatal aortic development. This intervention induced dilation and histological abnormalities in the aortic root, with minor involvement of the ascending aorta. To analyze early and late events associated with disease progression, we performed a comparative single cell transcriptomic analysis at 10- and 18-weeks post-deletion, when aortic dilation is undetectable and moderate, respectively. At the early time-point, *Smad3*-inactivation resulted in a broad reduction in the expression of extracellular matrix components and critical components of focal adhesions, including integrins and anchoring proteins, which was reflected histologically by loss of connections between VSMCs and elastic lamellae. At the later time point, however, expression of several transcripts belonging to the same functional categories was normalized or even upregulated; this occurred in association with upregulation of transcripts coding for TGF-β ligands, and persistent downregulation of negative regulators of the pathway. To interrogate how VSMC heterogeneity may influence this transition, we examined transcriptional changes in each of the four VSMC subclusters identified, regardless of genotype, as partly reflecting the proximal-to-distal anatomic location based on *in situ* RNA hybridization. The response to *Smad3*-deficiency varied depending on subset, and VSMC subsets over-represented in the aortic root, the site most vulnerable to dilation, most prominently upregulated TGF-β ligands and pro-pathogenic factors such as thrombospondin-1, angiotensin converting enzyme, and pro-inflammatory mediators. These data suggest that *Smad3* is required for maintenance of focal adhesions, and that loss of contacts with the extracellular matrix has consequences specific to each VSMC subset, possibly contributing to the regional susceptibility to dilation in the aorta.

## Introduction

Loeys-Dietz syndrome (LDS) is an autosomal dominant connective tissue disorder characterized by skeletal, ocular, and cardiovascular manifestations including highly penetrant and aggressive aneurysms (1, 2). LDS is caused by heterozygous, inactivating mutations in positive regulators of the TGF-β signaling pathway (1–6). TGF-β ligands bind to TGF-β receptor type II, which recruits and phosphorylates TGF-β receptor type I (TβRI). Upon activation, TβRI phosphorylates the receptor regulated Smad proteins, Smad2 and Smad3. Phosphorylated Smad2 and Smad3 can then bind to Smad4 and translocate to the nucleus, where they regulate the expression of TGF-β responsive genes (7). Heterozygous loss-of-function mutations in the ligands, receptors, and positive intracellular signaling mediators have been associated with LDS and result in an array of phenotypic manifestations, typically including aggressive aortic aneurysm (8). These include mutations in *SMAD3*, the vast majority of which are missense or frameshift mutations in the MH2 domain (9), which disrupt its ability to interact with both effectors and regulators, including the TGF-β receptors and Smad4 (10). This domain is essential for Smad-dependent transcriptional regulation and loss of function mutations in this region result in impaired transcriptional response to TGF-β signaling (11). Complete abrogation of TGF-β signaling in vascular smooth muscle cells (VSMCs), either prenatally or postnatally, is severely deleterious to aortic wall homeostasis (12–14) and results in mechanical failure and dissections in mouse models. However, in both LDS and other familial non-syndromic forms of aortic disease caused by mutations in positive effectors of the pathway (15), signaling output is only partly impaired, leaving open the possibility that compensatory mechanisms may restore or even upregulate signaling output in specific *in vivo* contexts, which is consistent with observations made in both patients and mouse models (1–5, 16, 17). The timing, the cell type-specific consequences, and the adaptive or maladaptive nature of this secondary response during disease progression remains unclear (18). In this study, we examine the aortic phenotype and smooth muscle-specific transcriptional changes in a mouse model with bi-allelic smooth muscle-specific postnatal *Smad3* deletion. In order to assess whether aortic dilation, and associated compensatory signaling upregulation, could be established independently of developmentally sensitive processes, bi-allelic *Smad3* deletion was induced at 6-weeks of age, a time when perinatal matrix deposition and associated phenotypic changes is considered to be completed (18, 19). In addition, we used this controllable system to assess both early and late transcriptional changes that occur *in vivo* upon *Smad3* inactivation and examined the smooth muscle specific transcriptional consequences of this intervention at either 10- or 18-weeks post-deletion, when aortic root dilation is undetectable or moderate, respectively.

## Materials and Methods

### Animal Experiments

All animal experiments adhered to protocols approved by the Animal Care and Use Committee at Johns Hopkins University School of Medicine. All mice were backcrossed to 129S6/SvEv mice (Taconic, 129SVE). Mice were housed in a pathogen-free facility with free access to standard chow and water, with a light/dark cycle of 10/14 h. Mice bearing *CAG-Sun1/sfGFP* (20) (The Jackson Lab, 021039) tracer allele with either a *Mef2c*^*SHF*^ (21) (gifted by the K.R. Chien lab at the Cardiovascular Research Center, Massachusetts General Hospital, Boston, Massachusetts, USA) or *Wnt1-Cre2* (22) (The Jackson Laboratory, 003829) were used for lineage tracing for bulk RNA- transcriptomic analysis. *Smad3*^+/+^ and *Smad3*^*lox*/*lox*^ (23) (a kind gift of Dr. Matzuk, Baylor College of Medicine) with *Myh11-Cre*^*ER*^ (24) (The Jackson Laboratory, 019079), some bearing the *EGFP-L10a* (25) (The Jackson Lab, 024750) conditional tracer allele, were injected with tamoxifen (2 mg/day) (MilliporeSigma, T5648) for five consecutive days starting at 6-weeks of age. As *Myh11-Cre*^*ER*^ is integrated on the Y chromosome, only male mice were used. No phenotypic differences were observed between mice with and without the conditional *EGFP-L10a* allele. *Smad3*^+/+^; *Myh11-Cre*^*ER*^mice are referred to as *Smad3*^+/+^, while *Smad3*^*lox*/*lox*^*; Myh11-Cre*^*ER*^ are referred to as *Smad3*^*SmKO*^, independently of the presence of the *EGFP-L10a* allele. Aortic dimensions of experimental mice were monitored by serial echocardiography using the Visual Sonics Vivo 2100 machine and a 30 mHz probe. Before being euthanized, tail cuff blood pressure measurements were taken for mice using the Visitech BP-2000 Non-Invasive tail cuff device. Mice were euthanized prior to tissue extraction using halothane (Millipore Sigma, H0150000) at a standard concentration (4%; 0.2 ml per liter of container volume).

### Validation of Smad3 Inactivation in *Smad3^*SmKO*^* Mice

Aortas were dissected, flash frozen, and stored at −80°C prior to protein isolation. Forty microliters of protein extraction buffer (Full Moon Biosystems, EXB100) containing phosphatase and protease inhibitor tablets (MilliporeSigma, 11836170001 and MilliporeSigma, 4906845001, respectively) and 8 lysis beads (Full Moon Biosystems, LB020) were added to each sample and protein was extracted using an MP Biomedicals FastPrep-24 5G automatic bead homogenizer. Immunoblot was performed using a 4–12% Bis-Tris Criterion XT gel (BioRad, 3450123) run for 1 h 30 mins at ~100 V (50 V for first 30 mins, then increased to 100 V for 30 mins, and 150 V for the final 30 mins). The gel was transferred to a PVDF membrane (BioRad, 1704273) using the BioRad Turbo Blot Transfer Standard 30-min protocol. The membrane was blocked for 45 mins in blocking buffer (LI-COR, 927-70003) diluted 1:1 with 1x PBS at room temperature. The following primary antibodies were used: anti-Smad3 from Invitrogen (MA5-14939), diluted 1:500 in blocking buffer, anti-beta-actin from Cell Signaling Technology (8H10D10) diluted 1:5000. The primary antibodies were diluted in blocking buffer and incubated for 72 h at 4°C. The membrane was washed 3 times for 5 mins in 1x PBS + 0.05% Tween20 (PBST). Appropriate secondary antibodies (LI-COR 926-68072 and 926-32213 diluted 1:10,000 in blocking buffer) were incubated in the dark at room temperature for 60 mins. The membrane was washed as before, 3 times in PBST, followed by 2 washes in 1x PBS before visualizing on the LI-COR Odyssey. For RNA assays, aortas were dissected and placed in 300 μl of TRIzol (ThermoFisher, 15596018) with 6 zirconium beads (OPS Diagnostics, BAWZ 3000-300-23). Samples were lysed using the MP Biomedicals FastPrep-24 5G automatic bead homogenizer. RNA was extracted and purified using the Direct-zol RNA MiniPrep kit (Zymo Research, R2052) according to the manufacturer's instructions. Complementary DNA (cDNA) was generated using the High-Capacity cDNA Reverse Transcription kit (Applied Biosystems, 4368813) as per the manufacturer's protocol. The qPCR was performed using the TaqMan Gene Expression Master Mix (Applied Biosystems, 4369016) and TaqMan probes (*Smad3* Mm01170760_m1, *Myh11* Mm00443013_m1) and run on the QuantStuido7 Flex with technical and biological replicates. Relative abundance of *Smad3* was obtained by normalizing against *Myh11* transcript abundance using the formula 2^−(Smad3Ct−Myh11Ct)^.

### Aortic Tissue Preparation and Histology

After euthanasia by halothane inhalation, the heart and thoracic aorta were dissected en bloc and fixed overnight at 4°C in 4% paraformaldehyde (Electron Microscopy Sciences, 15710) in PBS as previously described (16). Samples were then stored overnight at 4°C in 70% ethanol and mounted in 2% agarose in deionized water prior to paraffin embedding. Paraffin blocks were cut into five-micron sections, exposing a longitudinal cross-section of the heart and thoracic aorta. These sections were stained with Verhoeff-van Gieson (VVG) (StatLab, STVGI) to visualize elastic fiber morphology or used for *in situ* methods as described below.

### Electron Microscopy

After euthanasia by halothane inhalation, the heart and thoracic aorta were dissected en bloc and fixed overnight at 4°C in a solution of 0.1M Sodium Cacodylate and 4% paraformaldehyde (Electron Microscopy Sciences, 15710) in deionized water. The next day, samples were transferred to a solution of 30% sucrose and incubated overnight at 4°C. Samples were then embedded in OCT compound (Sciegen, 4586). Each sample was first bathed in OCT then perfused with 50% OCT in deionized water through the left ventricle. Samples where then embedded in OCT in plastic molds on a bed of liquid nitrogen and dry ice and placed at −80°C overnight to freeze completely. OCT blocks were sectioned using a Cryostat to produce 20-micron sections exposing a longitudinal cross-section of the heart and thoracic aorta. Selected slides were fixed with 2.5% Glutaraldehyde followed by 2% Osmium in Cacodylate buffer. Sections were embedded in EPON resin and trimmed to the appropriate region (aortic root or ascending aorta) before sectioning. Sixty nm sections of aortic root or ascending aorta were triple stained with tannic acid, uranyl acetate, and lead citrate. Images were acquired at 120 Kv on a Thermo-Fisher Talos L120C G2 Electron microscope with a 16-megapixel CMOS camera.

### Analysis of Smooth Muscle Cell-Elastic Fiber Contacts

For each animal, at least 14 regions were analyzed using FIJI (26). The length of visible elastic fibers was measured using the line tool, and cell protrusions less than 50nm from elastin were counted manually by a blinded researcher. The total number of protrusions counted were normalized to the total measured elastic fiber's length for each animal.

### Fluorescence *in situ* Hybridization With RNAscope Probes

Paraffin embedded sections of *Smad3*^+/+^ and *Smad3*^*SmKO*^aortas were deparaffinized and prepared for RNA *in situ* hybridization using RNAscope probes from Advanced Cell Diagnostics. RNAscope Multiplex Fluorescent Reagent Kit v2 Assay (Catalog No. 323100) was performed as described in the manufacturer's protocol optimized for fixed samples. Probes used included *Mm-Enpep* (862211), *Mm-Mgp* (463381)*, Mm-Notch3* (425171)*, Mm-Ptprz1* (460991), and *Mm-Tes* (1092801). Images were acquired on a Zeiss LSM880 Airyscan FAST confocal microscope at 20× magnification. Images are presented as maximal intensity projection and equally adjusted across samples to enhance visualization of information present in the original.

### Aortic Dissociation and Single Cell RNA Sequencing

The following protocol for aortic dissociation was adapted from published protocols (27, 28). The aorta was perfused with 5 ml of PBS injected through the left ventricle of the heart. The thoracic aorta from the aortic root to the arch (sinus of Valsalva to the branchpoint of the left subclavian artery) was collected and cut into small pieces and placed into 500 μl of solution A [4.5 ml HBSS (Thermo Fisher Scientific, 14175095), 500 μl 50% D-Trehalose (Quality Biosciences, A611-K480-95), 45 μl RNase inhibitor [20 U/μl] (Applied Biosystems, N8080119)], all at room temperature. One microliter of DyeCycle Violet (DCV) (ThermoFisher, V35003) was added to stain double stranded DNA. After all aortic samples were collected, the tissue was enzymatically digested by adding 500 μl of solution B (5 ml of HBSS, 8 mg Collagenase II) (315 U/mg) (Worthington-Biochem, LS004176), 100 μl of Elastase (MilliporeSigma, E1250), 1 mg of DNase I (2,610 U/mg) (Zymo Research, E1009) and incubating the sample in a thermomixer set to 800 rpm for 30 mins or 1 h at 37°C. After incubation, each sample was gently pushed through a 3 ml syringe with a 21G needle 8 times to mechanically disrupt the tissue. To inhibit the enzymes, 100 μl of 100% FBS (Corning, 35-010-CV) was added to each sample tube. The cells were pelleted at 800× g for 3.5 mins at room temperature. The supernatant was discarded, and cells were resuspended in 500 μl of solution A and 1 ul/ml of DCV. The cell suspension was carefully pipetted through a 40 μm filter cap tube. Propidium iodide (PI) (ThermoFisher Scientific, P1304MP) was added to each sample and cells were sorted by flow cytometry to produce a single cell suspension of DCV^+^, PI^neg^ cells. Twenty microliters of 1% BSA in HBSS was used as catch buffer. A single-cell suspension of roughly 3,000–8,000 live, nucleated aortic cells per mouse was obtained by Fluorescence Activated Cell Sorting (FACS) (the exact number of cells varied per sample), processed using the 10× Genomics Chromium 3' v3 platform, and sequenced on the Illumina NovaSeq sequencer at a mean depth of approximately 400 million reads per library (corresponding to the recommended 50,000/cell read depth). A total of 66,261 aortic cells from 16 mice were sequenced.

### Single Cell RNA Sequencing Data Analysis

Cell Ranger software (V6 from 10x Genomics) was used to process, align, and aggregate the raw data. Reads were mapped to the mouse mm10 genome that was modified to include the GFP sequence. The scRNA-seq data were filtered using the R package Seurat v4 (29) according to the following settings: >600 genes detected per cell but <6000, >2000 total molecules detected per cell but <50000, and <25% mitochondrial genes per cell. This filtering reduced the total cell number from 66,261 to 50,835 cells for further analysis. For clustering and cell-type specific analysis, the data were normalized using SCTransform, this function employs a negative binomial regression to normalize UMI count data. As samples were collected in several batches, the data were integrated using reciprocal PCA in Seurat (uses FindIntegrationAnchors function with k.anchors set to 20 and IntegrateData function). The data were then subjected to principal component analysis (RunPCA), uniform manifold approximation and projection (RunUMAP), and the FindNeighbors and FindClusters functions in Seurat. Clusters were visualized using the DimPlot function. For differential expression analysis, the raw data (RNA assay) were normalized and scaled using the NormalizeData (log transformation) and ScaleData functions. Cluster defining markers and differentially expressed genes between genotypes were found using the FindMarkers function which utilizes a Wilcoxon rank-sum test to identify differentially expressed genes between two groups of cells. Data were visualized using the DotPlot and FeaturePlot functions.

### Gene Over-representation Analysis

#### EnrichR Analysis of VSMC-Specific Transcriptional Changes

Analysis was conducted on 558 transcripts, filtered for detection in at least 10% of VSMCs at either 10- or 18-weeks post-deletion and an absolute logarithmic fold-change value (referred henceforth as Log_2_FC) of 0.2, and with a *P*-value below 0.05. After filtering of ribosomal and mitochondrial transcripts, 496 genes were submitted for gene over-representation analysis using EnrichR. A link to the full analysis, results and code can be found at this web address (https://maayanlab.cloud/Enrichr/enrich?dataset=e7883d5f07b8ca4112e10cdd8e32ebd6), courtesy of the Maayan lab cloud services (30–32).

#### REACTOME Functional Interaction Gene Network and Pathway Enrichment

The 558 transcripts detected in at least 10% of VSMCs at either 10- or 18- weeks post-deletion, with a minimum 0.2 Log_2_FC in either direction and a *P*-value below 0.05, were converted to their human orthologue, if available, for submission to the Pathway Enrichment analysis pipeline in the ReactomeFIVIz (33) package (https://reactome.org/tools/reactome-fiviz) using Cytoscape (34) (Version: 3.9.0). 540 transcripts were successfully mapped to a node, with 176 being unconnected nodes, which were excluded from further analysis. Non-mapped transcripts included gene segments, unmapped symbols, and non-protein coding/lncRNAs with no clear human orthologues. Cluster FI network and Analyze Module functions were used to identify ribosome and mitochondrial clusters, which were converted to single group node prior to executing the “Analyze Network Function, Pathway Enrichment” algorithm in ReactomeFIVIz. Relevant pathways identified by this analysis were visualized in the network using the Cytoscape “bypass” function to manually attribute specific fill-colors to nodes belonging to specific pathways.

#### Visualization of Expression Data in Pathways and Networks

Normalized expression data was imported as a table in Cytoscape and used to determine nodes “fill color” based on the value of average Log_2_FC of experimental samples relative to controls, according to the displayed legend. PathVisio 3.3.0 (35) was used to download and modify WikiPathways (36) of interest. The following WikiPathways were used to generate a merged a custom pathway: www.wikipathways.org/instance/WP523; www.wikipathways.org/instance/WP6; www.wikipathways.org/instance/WP85; www.wikipathways.org/instance/WP113. A similar process was used to overlay expression data on a merged diseased network downloaded from STRING Disease database for LDS1 to 5 (37).

#### ClueGO and CluePedia Analysis

The ClueGO (38) and CluePedia (39) pipeline was used for analysis of both global (only one list uploaded for all VSMCs) and subset-specific (four lists corresponding to each of the four VSMC subsets) transcriptional alterations caused by *Smad3* deletion, using the appropriate settings for absolute and relative gene enrichment. In either case, the gene list was compared against four ontologies: GO Biological Function, KEGG, Reactome, and WikiPathways; ribosomal and mitochondrial transcripts were filtered prior to gene enrichment analysis. ClueGO log files for each analysis are provided as Supplementary Material. The individual analysis of VSMC-wide changes was performed on the subset of ~200 misregulated transcripts that displayed an opposite trend of dysregulation at 10- and 18-weeks post-deletion. Subset-specific analysis was conducted on the most dysregulated genes in each subset (regardless of direction) that had Log_2_FC of at least 0.25 and a nominal *P*-value of 0.01 after processing in Seurat.

### Bulk RNA-Sequencing of Lineage-Sorted VSMCs

Mice bearing a conditional *CAG-Sun1/sfGFP* (20) (The Jackson Lab, 021039) tracer allele with either a *Mef2c*^*SHF*^ (21) (gifted by the K.R. Chien lab at the Cardiovascular Research Center, Massachusetts General Hospital, Boston, Massachusetts, USA) or *Wnt1-Cre2* (22) (The Jackson Laboratory, 003829) allele were sacrificed at 8 weeks of age. The aortic root and ascending aorta were dissected and cleaned with PBS. Minced tissue was transferred to ice-cold HBSS (Thermo Fisher Scientific, 14175095) containing 0.2 U/μl RNase inhibitor (Applied Biosystems, N8080119) and 5% D-trehalose (Quality Biosciences, A611-K480-95) and then digested with 1 mg/ml collagenase II (Worthington-Biochem, LS004176), 100 ul elastase (MilliporeSigma, E1250), and 0.2 mg/ml DNase I (Zymo Research, E1009) in a 37°C rotating water bath for 30 mins with periodic agitation. After further trituration through a 21G needle, the digestion was stopped by addition of 10% Fetal Bovine Serum (FBS) (Corning, 35-011-CV). Cells were pelleted by centrifugation, resuspended in the previously mentioned HBSS solution, and passed through a 40 μm filter-cap tube. Cells were sorted using a DakoCytomation MoFlo at the Bloomberg Flow Cytometry and Immunology Core. Viability was determined by propidium iodide stain and GFP^+^ cells were sorted into a tube containing TriZol (ThermoFisher Scientific, 15596026). After separation by chloroform and addition of cold 70% ethanol, RNA was extracted using the PicoPure RNA Isolation Kit (ThermoFisher Scientific, KIT0204). Total bulk RNA was normalized according to sorted cell number, reverse transcribed and amplified according to the SmartSeq2 protocol used for single cell sequencing (40). The amplified cDNA was fragmented and barcoded using the Nextera XT DNA Library Preparation Kit (Illumina, FC-131-1024) and the resulting libraries were pooled and sequenced using an Illumina HiSeq2500.

### Bulk RNA-Seq Data Analysis

RSEM (v1.2.25) (41) was used to align and create gene and isoform level expression estimates for genes in the mouse genome (10 mm). The function rsem-calculate-expression, using bowtie2 as the aligner, created a series of matrices of expression values that were then compared across groups and compiled into a single matrix. A matrix of gene level expression estimates (TPM), generated through RSEM, was filtered to identify transcripts that had an exclusive pattern of expression across all 3 replicates of either lineage, with at expression threshold of 5 TPM across the expressing lineage. This list of genes and associated expression values was uploaded in iDEP (42) for visualization of normalized expression as a heatmap.

### Statistical Analysis

#### Single Cell RNA Sequencing

The FindMarkers function in Seurat utilizes the Wilcoxon rank-sum test to determine significantly different transcripts between two groups of cells (i.e., clusters and/or genotypes). The adjusted *P*-value indicated in supplementary lists of cluster-defining transcripts and differentially expressed transcripts between genotypes is calculated using Bonferroni correction based on the total number of genes in the dataset.

#### All Other Experiments

For all other data analysis, statistical analysis and data visualization were performed in Graphpad Prism 9.0. Data were subjected to the Shapiro-Wilk test for normal distribution and F-test for equal variance. Upon verification of normal distribution and equal variance, control and experimental measurements were compared using unpaired, two-tailed Student's t-test. Unless otherwise indicated in figure legend, measurements are presented as “Box and whiskers,” with the whiskers indicating the minimum and maximum and dots representing all data points; horizontal bar in the box plot indicates the median. For comparison of growth curves, data was interpolated with a simple linear regression slope, which were then compared using the “Test whether slopes and intercepts are significantly different” function in Graphpad Prism 9.0. Whenever statistical significance is indicated in a figure, the statistical tests used are included in the figure caption.

## Results

### Postnatal *SMAD3* Deletion Induces Dilation and Histological Abnormalities That Preferentially Affect the Aortic Root

**Figure 1:**
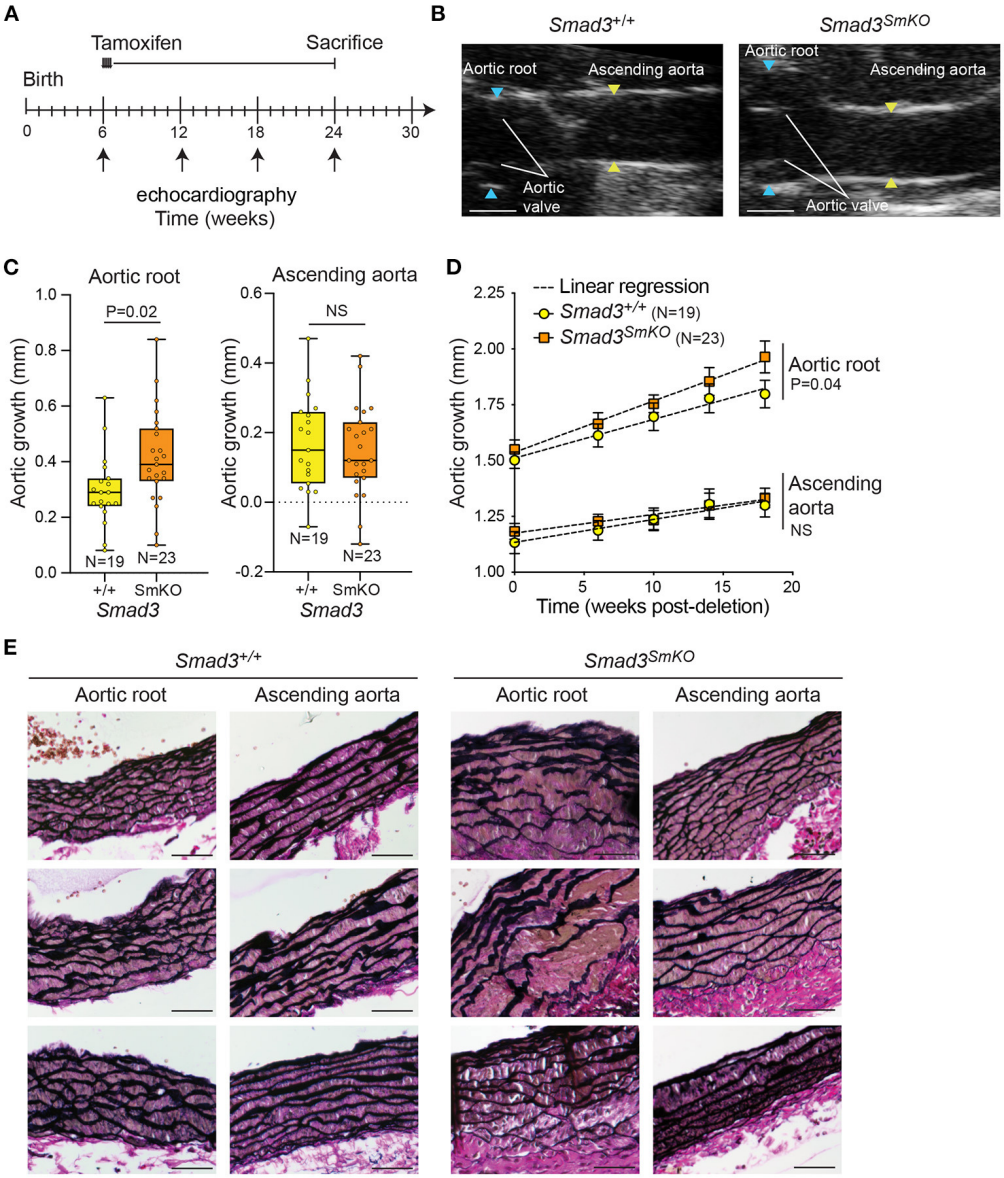
Postnatal deletion of *Smad3* in VSMCs induces significant dilation of the aortic root but not ascending aorta. **(A)** Schematic of postnatal *Smad3* deletion in smooth muscle cells using *Myh11-Cre*^*ER*^. Tamoxifen was administered at 6 weeks of age and mice were sacrificed at 24 weeks of age. Tamoxifen*-*treated *Smad3*^*flox*/*flox*^*;Myh11-Cre*^*ER*^ are referred to as *Smad3*^*SmKO*^, while Tamoxifen*-*treated *Smad3*^+/+^*;Myh11-Cre*^*ER*^ are indicated as *Smad3*^+/+^. **(B)** Representative echocardiographic images of *Smad3*^+/+^ and *Smad3*^*SmKO*^ aortas at 24 weeks of age, blue arrows indicate aortic root dimensions and yellow arrows indicate dimensions of the ascending aorta. Scale bar is 1 mm. **(C)** Aortic root and ascending aortic growth of control and *Smad3*^*SmKO*^ mice from 6 to 24 weeks of age as measured by echocardiography. *P*-value refers to two tailed Student's t-test. **(D)** Aortic root and ascending aortic size of *Smad3*^*SmKO*^ mice measured by echocardiography at baseline and several intervals post-deletion. The dotted line indicates simple linear-regression, and *P*-value refers to an analysis of covariance comparing the two slopes conducted on Graphpad. **(E)** Representative sections of the aortic root and ascending aorta obtained from *Smad3*^+/+^ and *Smad3*^*SmKO*^ animals and stained with Verhoeff Van Gieson; three individual biological replicates per condition are shown. Scale bar is 50 μm.

To examine the VSMC-specific impact of postnatally impaired TGF-β/Smad signaling in a mouse model in which time-sensitive transcriptional changes could be examined, we bred conditional *Smad3*^*flox*/*flox*^ mice to transgenic mice carrying a tamoxifen-inducible *Cre* recombinase that is specifically expressed in VSMCs (*Myh11-CRE*^*ER*^) (23). *Smad3*^*flox*/*flox*^and *Smad3*^+/+^control mice were treated with tamoxifen at 6 weeks of age (Figure 1A). Mice in which *Smad3* is specifically deleted in VSMCs are referred to as *Smad3*^*SmKO*^, and controls as *Smad3*^+/+^. Smad3 protein and RNA expression was significantly reduced in the aortas of *Smad3*^*SmKO*^ mice relative to controls (Supplementary Figure 1). The aortas of control and *Smad3*^*SmKO*^ mice were monitored by serial echocardiography from 6 to 24 weeks (Figure 1B). This intervention was sufficient to induce significant dilation of the aortic root, but not the ascending aorta by 24 weeks of age (18 weeks post-deletion) (Figures 1C,D). Aortic dilation was associated with defective aortic wall architecture, which was also more prominent in the aortic root relative to the ascending aorta of the same *Smad3*^*SmKO*^ animal (Figure 1E). A smaller cohort of heterozygous *Smad3*^*flox*/+^*; Myh11-CRE*^*ER*^ mice was also analyzed; however, these animals did not develop aortic dilation and were thus not included in further experiments. Dilation of the aortic root in response to *Smad*3 deletion occurred with no systemic changes in blood pressure when compared to controls at 24 weeks of age (Supplementary Figure 2).

### Single Cell Analysis of Control and *Smad3*-Deficient Mice at 10- and 18-Weeks Post-deletion

**Figure 2:**
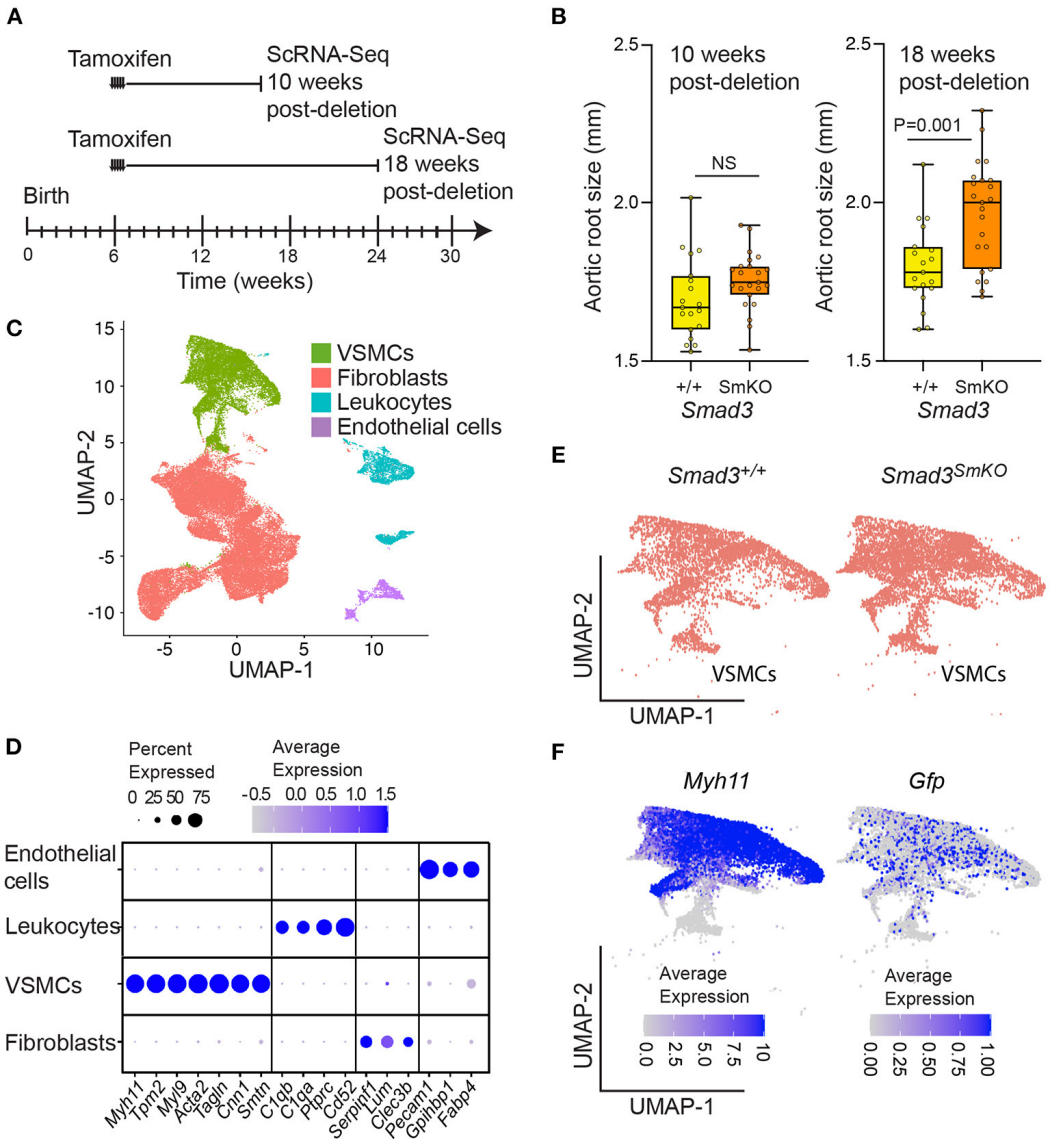
Single cell transcriptomic analysis of control and *Smad3*^*SmKO*^ mice at 10- and 18-weeks post-deletion. **(A)** Schematic of postnatal *Smad3* deletion by tamoxifen administration at 6 weeks of age and aortic tissue collection for single cell RNA sequencing at 10- and 18-weeks post-deletion. **(B)** Aortic root size of *Smad3*^*SmKO*^ mice at 10- and 18-weeks post-deletion as measured by echocardiography. *P*-value refers to two tailed Student's *t*-test. **(C)** Uniform manifold projection and approximation (UMAP) of aortic cells from *Smad3*^+/+^ and *Smad3*^*SmKO*^ mice. **(D)** Dot plot showing lineage specific transcripts used to identify vascular smooth muscle cells (VSMCs), fibroblasts, endothelial cells, and leukocytes. The intensity of the dot's color corresponds to a scaled average expression of each transcript while the size of the dot indicates the percentage of cells in each cluster that express that transcript. **(E)** UMAP showing the distribution of VSMCs in *Smad3*^+/+^ and *Smad3*^*SmKO*^ mice. **(F)** UMAPs showing the distribution of *Myh11*- and *Gfp-*expressing cells within the VSMC cluster. Mice with a *Gfp* lineage-tracer induced by *Myh11-Cre*^*ER*^ were included in this analysis, as such cells expressing *Gfp* are those that were marked by *Myh11-Cre*^*ER*^ at the time of tamoxifen induced Cre-mediated recombination.

In order to investigate the transcriptional changes associated with aortic dilation in *Smad3*^*SmKO*^ mice and assess how they may be affected by timing and cellular context, we performed single cell RNA transcriptomic analysis on thoracic aortas of control and *Smad3*^*SmKO*^ mice at both 10- and 18-weeks post-deletion (Figure 2A; Supplementary Figure 3), when aortic root dilation is undetectable or moderate (Figure 2B). The segment of aorta dissected for this analysis included the aortic root, proximal and distal ascending aorta as well as the aortic arch, up to the boundary defined by the left subclavian artery. All major aortic cell types were identified using previously defined (27) lineage-specific transcripts (Figures 2C,D). We identified the same population of VSMCs regardless of genotype (Figure 2E). In mice in which VSMCs were traced by expression of a conditional fluorescent reporter, the VSMC cluster included cells expressing the associated transcript (although its expression was low), indicating that these cells expressed the VSMC-specific Myosin Heavy Chain 11 (*Myh11*)-promoter driven *Cre* recombinase at the time of tamoxifen administration (Figure 2F). To investigate the effect of VSMC-specific *Smad3* deletion overtime we performed differential expression analysis to identify significant differences in transcript expression between control and *Smad3*^*SmKO*^ mice at 10- and 18-weeks post deletion (Supplementary Tables 1, 2). 548 transcripts detected in at least 10% of cells, with an average Log_2_FC of 0.2 (in either direction) and an adjusted *P* value below 0.05, at either time-point were selected for further analysis (Supplementary Table 3).

### Defective Expression of TGF-β-Induced Focal Adhesion and Extracellular Matrix Components Precedes Detectable Aortic Dilation After Postnatal VSMC-Specific *Smad3* Deletion

**Figure 3:**
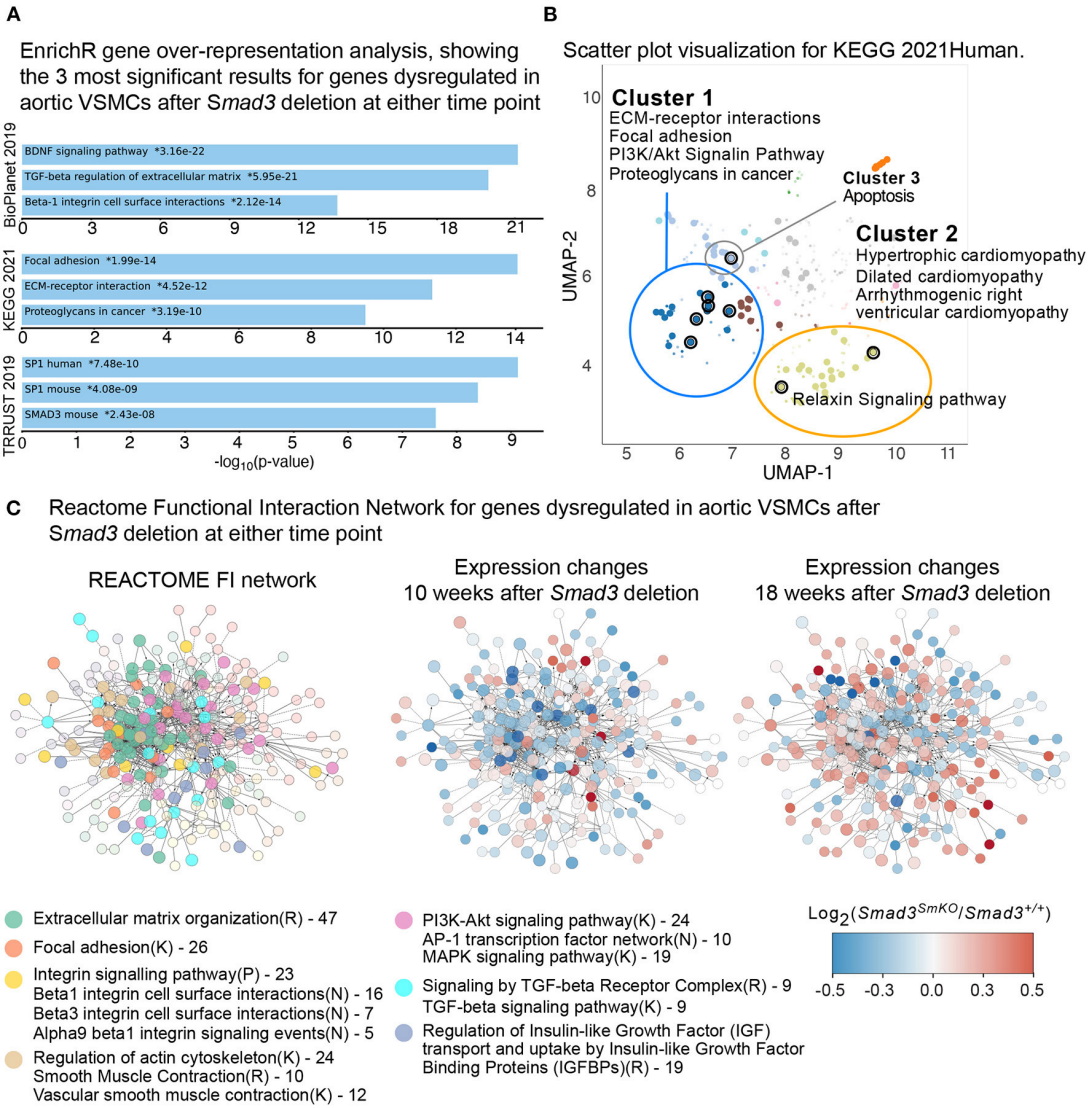
Defective expression of extracellular matrix and focal adhesions components in *Smad3*-deficient VSMCs. **(A)** EnrichR gene over-representation analysis, showing the top 3 most significant results in the BioPlanet 2019, KEGG 2021, and TRRUST 2019 databases for genes dysregulated in aortic VSMCs after S*mad3* deletion at either time point. **(B)** Scatter plot visualization of top dysregulated KEGG 2021 pathways in VSMCs after S*mad3* deletion. Similar gene sets are clustered together, and larger, black-outlined points represent significantly enriched terms. Clusters were computed using the Leiden algorithm and points plotted on the first two UMAP dimensions by the EnrichR web application. **(C)** Dysregulated transcripts were converted to their human orthologue and analyzed using the REACTOME Functional Interaction (FI) Gene Set enrichment pipeline, which yielded a main network of approximately 300 transcripts. Each node represents a dysregulated gene in *Smad3-*deficient VSMCs. Nodes were manually colored-coded based on the main enriched pathway they belonged to, as indicated in the legend. R indicates a REACTOME pathway, K a KEGG and N and NCI Pathway Interaction Database; numbers listed in the legend represent number of transcripts belonging to the indicated group. The same network of dysregulated genes is also shown computationally colored based on corresponding transcript expression at 10- and 18-weeks post-deletion. Genes shown in blue are downregulated and genes shown in red are upregulated in *Smad3*^*SmKO*^ relative to controls.

Gene enrichment analysis was performed using EnrichR (30–32), selecting BioPlanet2019 (43), Kyoto Encyclopedia of Genes and Genomes (KEGG) (44), and TTRUST (45) as the databases for comparison. Major pathways significantly dysregulated in *Smad3-*deficient VSMCs included, as would be expected, those related to TGF-β regulation of the ECM, as well as to *Smad3*-transcriptional regulation. Brain-Derived Neurotrophic Factor (BDNF) signaling, focal adhesions, regulation of the actin cytoskeleton, and ECM-receptor interaction were also perturbed (Figures 3A,B).

Additional pathways identified as dysregulated included those related to cardiomyopathy, with gene overlap including *Tgfb2, Thbs1* (thrombospondin-1, also known as Tsp-1), and *Ace* (Angiotensin I Converting Enzyme, or ACE), all of which code for factors previously implicated in both vascular and cardiac pathogenesis (46). In particular, both ACE, which is a positive modulator of Angiotensin II signaling, and Thbs1, a multifunctional protein with both pro-inflammatory and cell signaling regulatory functions, have been shown to be upregulated in aneurysm tissue and implicated in its pathogenesis (46). Components of the Relaxin pathway, which signals primarily through its G protein coupled receptor and has been shown to have cardioprotective and anti-fibrotic effects, were also misregulated (47) (*Gng11* and *Adcy5*) (Figure 3B). The full analysis can be accessed at the link provided in the Materials and Methods Section.

In order to visualize network interactions between dysregulated transcripts, and changes in expression as attributes of specific nodes, we also performed a Functional Interaction (FI) gene enrichment analysis using the ReactomeFI (33) plug-in for the network analysis software Cytoscape (34).

**Figure 4:**
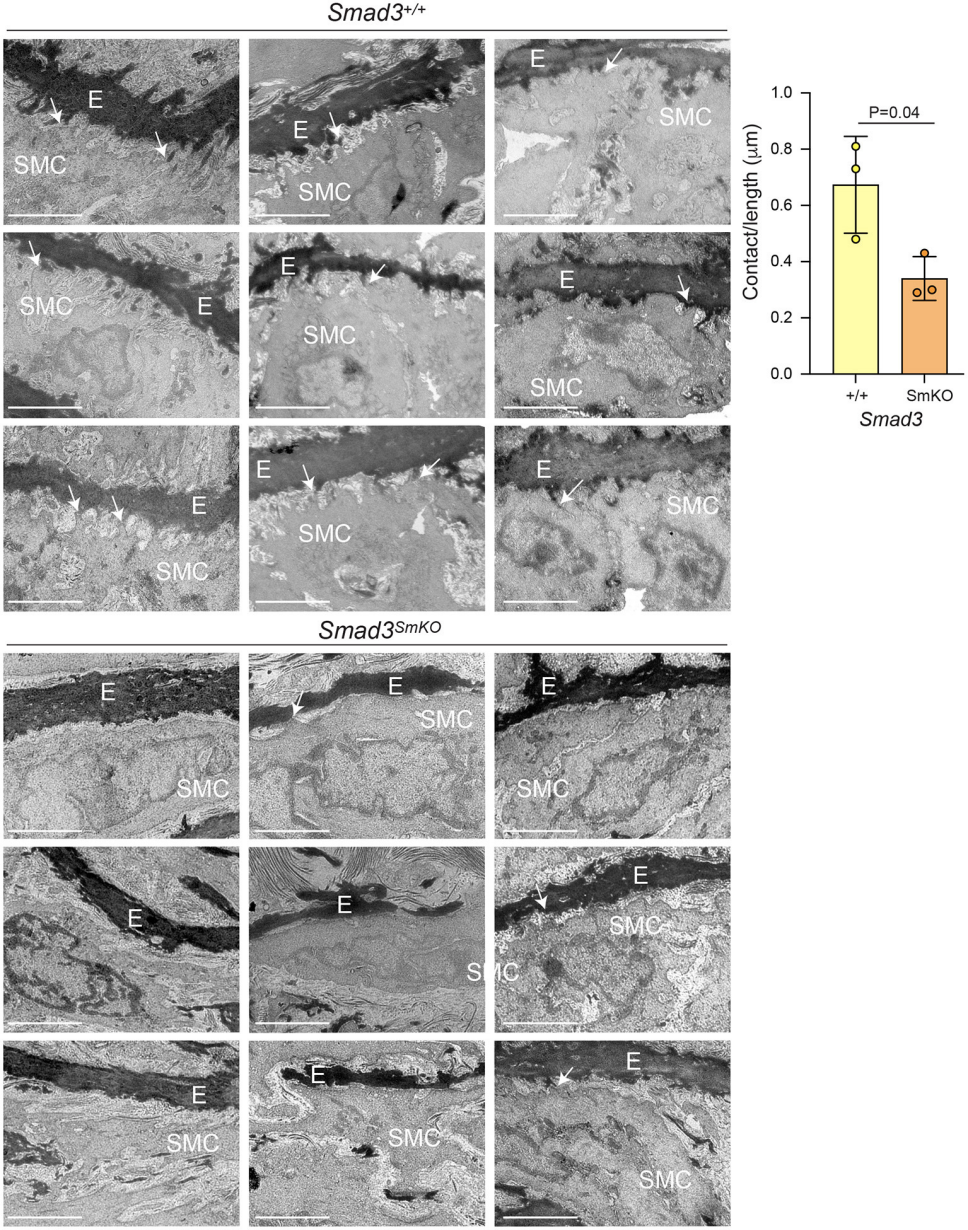
Transcriptional alterations associate with loss of connections between VSMCs and the elastic lamellae *in vivo*. Representative electron micrographs of longitudinal sections of the aortic root of *Smad3*^+/+^ and *Smad3*^*SmKO*^ mice 10 weeks after *Smad3* deletion (E, elastic lamellae; SMC, Smooth muscle cell, arrows indicate contact between SMCs and the ECM, note only some connections are labeled to help orient the reader to this feature). Three biological replicates, with three representative images from each animal, per condition are shown. Scale bar is 2 μm. Cell protrusions <50 nm from elastin were counted manually by a blinded researcher. The total number of protrusions counted were normalized to the total measured elastic fiber's length for each animal (Contact/length). *P*-value refers to two tailed Student's t-test.

This analysis also showed widespread perturbation of the pathways related to ECM organization, focal adhesions, integrins, smooth muscle contraction and TGF-β signaling (Figure 3C). A closer investigation of VSMC-specific transcriptional differences between control and *Smad3*^*SmKO*^mice 10 weeks after *Smad3* deletion shows that an early event in the pathogenesis of aortic dilation, and which occurs before aortic dilation is detectable by echographic assessment, is impaired expression of transcripts encoding for adaptor proteins and other actin-binding proteins that connect integrins to the actin cytoskeleton thorough focal adhesion (*Fblim1, Tln1, Vcl, Lims2, Actn1*, and *Flna*) (Supplementary Figure 4). Additionally, expression of integrins (*Itga1, Itga7, Itga8*, and *Itga9*), elastic fiber components (*Eln, Fbln5*, and *Mfap4*), and some but not all smooth muscle contractile genes (*Cnn1, Myh11, Mylk*, and *Myocd*) was also impaired (Supplementary Figure 4). Taken together these data suggest that assembly of focal adhesions in *Smad3*-deficient VSMCs may be defective because of decreased availability of both extracellular and intracellular components, and because of defective force-dependent maturation, which depends on myosin II activity (48). Accordingly, examination of aortic architecture 10 weeks after *Smad3* deletion by electron microscopy shows that *Smad3*-deficient VSMCs form fewer connections and/or lose contact with the elastic lamellae as compared to controls (Figure 4). These early transcriptional changes were also accompanied by decreased expression of negative regulators of TGF-β (*Pmepa1, Skil, Nr4a1, Wwp2*) all of which participate in negative feedback control of TGF-β signaling by being both target genes and inhibitors of the pathway (49–52) (Supplementary Figure 4).

### Detectable Aortic Dilation Is Associated With Restoration of Normal Expression and/or Upregulation of Selected Smad-Dependent Transcripts

The downregulation of transcripts coding for inhibitors of TGF-β/Smad signaling may partly explain why analysis of the expression differences that occur 18 weeks after *Smad3*-deletion showed a substantial reversal in the direction of change for several transcripts. Although the functional categories of dysregulated transcripts did not vary with time-point analyzed, many transcripts downregulated at the 10-week time point showed either normal expression, or even upregulation, at the 18-week time point (Supplementary Figure 4). Approximately 200 transcripts among those detectable by scRNA sequencing displayed time-sensitive transcriptional deregulation upon *Smad3* deletion. These data suggested that the response of VSMCs to impaired *Smad3* signaling is dynamic and that compensatory mechanisms, presumably acting on the remaining functional branches of the pathways such as those activated by TGF-β through Smad2, can modify the effect of the initial signaling loss.

**Figure 5:**
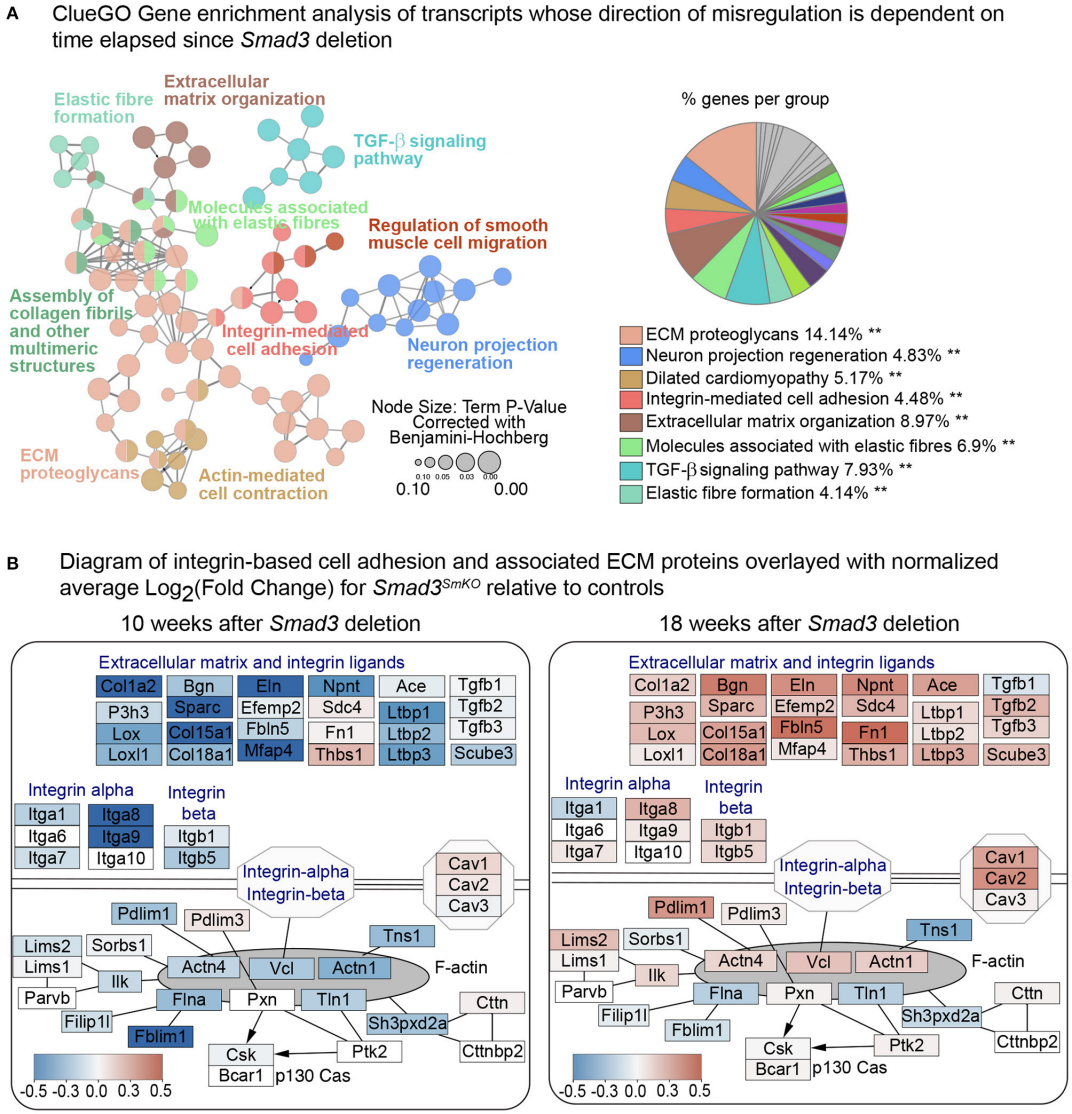
*Smad3* deletion causes time-sensitive defects in expression of focal adhesion and ECM components. **(A)** ClueGO Gene enrichment analysis of transcripts whose direction of change in *Smad3*^*SmKO*^ relative to controls is dependent on time elapsed since *Smad3* inactivation. 153 transcripts had a negative Log_2_FC 10 weeks after *Smad3* deletion, but a positive Log_2_FC at 18 weeks, and 105 showed the opposite behavior, for a total of 258 transcripts displaying a time-sensitive transcriptional dysregulation upon *Smad3* deletion. Each node represents a group of genes sharing a similar function. The color of the node corresponds to a ClueGO functional group to which each node belongs. Nodes belonging to more than one ClueGO functional group are filled with multiple colors. Asterisks indicate significance as calculated by ClueGO algorithm and corrected using the Benjamini-Hochberg method for multiple testing correction. **(B)** Custom pathway diagram adapted from WikiPathways WP523, WP6, WP85 and WP113 using the PathVisio 3.0 software, and overlayed with relative expression data using Cytoscape. Each node shows expression of transcripts coding for extracellular matrix components, integrin ligands, integrins, and intracellular focal adhesion components in VSMCs 10 weeks and 18 weeks after *Smad3* deletion. Genes shown in blue are downregulated and genes shown in red are upregulated in *Smad3*^*SmKO*^ relative to controls. Scale indicates the average Log_2_FC in expression.

To perform a gene enrichment analysis across multiple databases (Gene Ontology -Biological function, KEGG, Reactome and WikiPathways), and visualize the resulting enriched pathways as a functional network, this smaller list of genes was analyzed using ClueGO (38). ClueGO organizes enriched pathways and terms into “ClueGO groups” according to shared number of genes and/or terms related to a shared biological meaning; enriched pathways are then visualized in a network where each node represents a group of genes sharing a similar function (38, 39). Transcripts displaying time-sensitive transcriptional changes after *Smad3*-deletion in VSMCs represent all the major pathways known to be involved in the pathogenesis of aortic vasculopathies (15, 53), with a preponderance of transcripts being involved in cell-matrix interactions (Figures 5A,B). The results of this analysis are provided in Supplementary Table 4. This transcriptomic analysis suggests that widespread disruption of genes associated with the extracellular matrix and smooth muscle mechanosensing occurs early after *Smad3* inactivation, while the onset of dilation is marked by potentially adaptive or maladaptive increases in expression of selected extracellular matrix and focal adhesions components, presumably through compensatory activation of Smad2 and other parallel pathways.

### VSMC Subclusters Correlate With Proximal to Distal Location in the Murine Aorta

**Figure 6:**
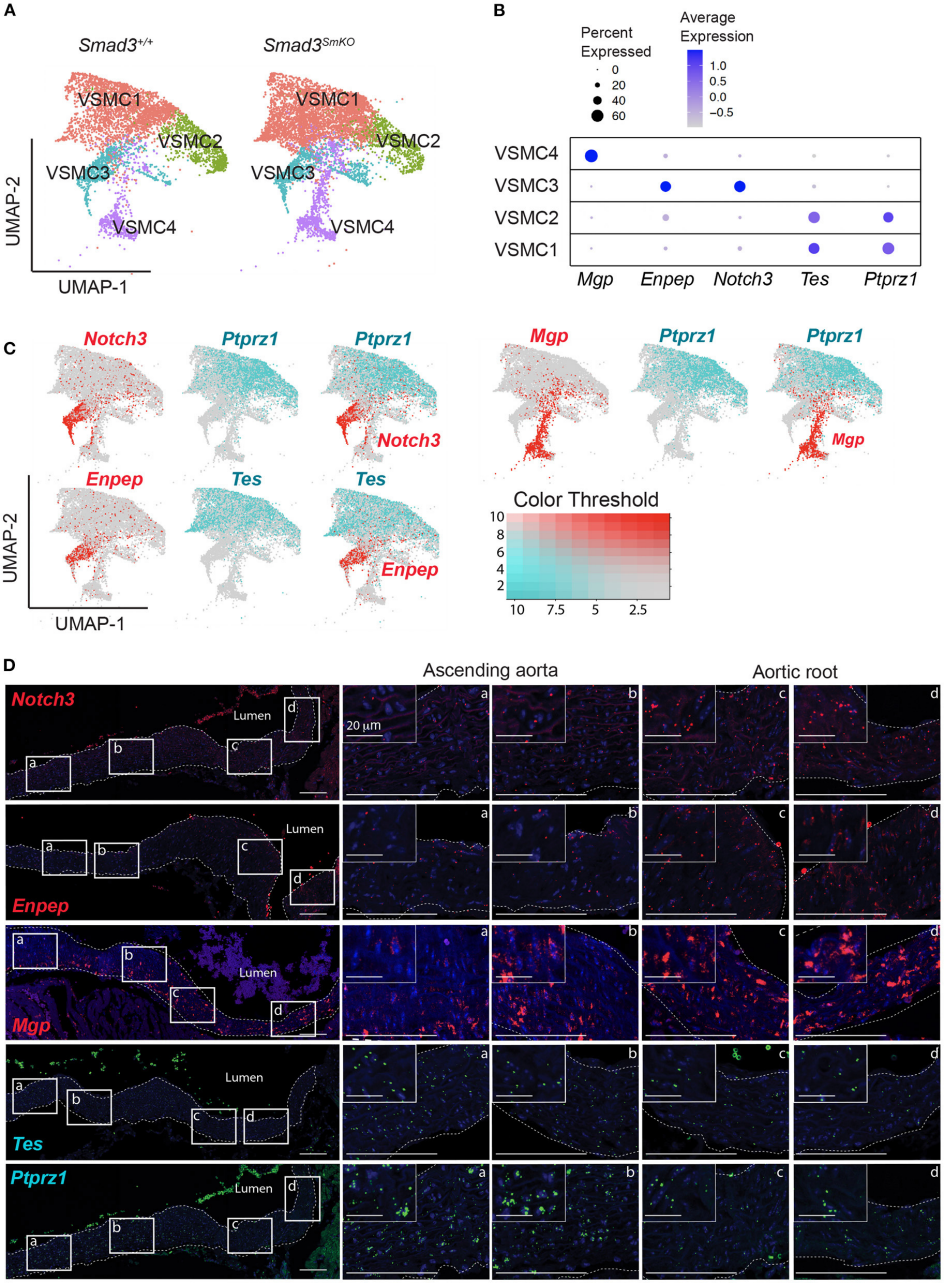
Single cell transcriptomic analysis reveals four subpopulations of VSMCs and a gradient of cluster-defining transcripts that define the proximal to distal location in the murine aorta. **(A)** Uniform manifold approximation and projection (UMAP) of vascular smooth muscle cells (VSMCs) from *Smad3*^+/+^ and *Smad3*^*SmKO*^ aortas reveals four subclusters irrespective of genotype. **(B)** Dot plot showing expression of cluster-defining transcripts for the VSMC subclusters. The intensity of the dot's color corresponds to a scaled average expression of each transcript while the size of the dot indicates the percentage of cells in each cluster that express that transcript. **(C)** Feature plots showing the distribution of *Mgp, Enpep, Notch3, Tes*, and *Ptprz1* expressing-cells across all VSMC clusters. **(D)** Representative images of RNA fluorescence *in situ* hybridization for *Notch3, Enpep, Mgp, Tes*, and *Ptprz1* in 16-week-old control aortas. Insets identify locations shown at higher magnification in subsequent panels. Images were acquired as a tile with x20 magnification. Scale bars are 100 μm and 20 μm as indicated on figure.

To better understand the contribution of cellular heterogeneity to the global changes observed in *Smad3*^*SmKO*^ VSMCs, we investigated the substructure of this cluster. Four VSMC clusters were identified regardless of genotype (Figure 6A), as determined based on the distribution of cluster-defining transcripts (Supplementary Table 5). Cluster-defining transcripts included *Mgp*, which was enriched in VSMC cluster 4, *Notch3*, and *Enpep* enriched in VSMC cluster 3, and *Ptprz1* and *Tes*, enriched in VSMC clusters 1 and 2 (Figure 6B). Visualization of the distribution of cells expressing these cluster-defining transcripts in all VSMCs (*Notch3* vs. *Ptprz1, Mgp* vs. *Ptprz1*, and *Enpep* vs. *Tes*), showed very little overlap in cells expressing *Mgp, Notch3 or Enpep* and cells expressing *Ptprz1* or *Tes* (Figure 6C). In order to test if these VSMC subclusters reflected the regional distribution along the aorta, we performed RNA *in situ* hybridization with probes specific for cluster-defining transcripts that showed the most consistent selective distribution across individual samples. Independently of genotype, we observed a gradient of expression for *Notch3, Mgp*, and *Enpep*, which were enriched in the aortic root and proximal ascending aorta and *Ptprz1* and *Tes*, which were enriched in the more distal ascending aorta (Figure 6D). Notably, while cells expressing *Notch3, Enpep*, and *Mgp* were all enriched in the aortic root, cells expressing these transcripts showed little overlap by UMAP, suggesting that transcriptional differences beyond location further distinguish these cells.

The distal-to-proximal distribution of expression of cluster-defining transcripts for VSMC cluster 1 to cluster 4 was reminiscent of the distribution of cardiac neural crest (CNC)- and secondary heart field (SHF)-derived VSMCs in the aorta (54). Having obtained a list of lineage-exclusive transcripts from bulk RNA sequencing of lineage-traced aortic VSMCs of either lineage (Supplementary Table 6), we examined their distribution across the four VSMC subsets identified by scRNA sequencing analysis (Supplementary Figures 5A–D). Among transcripts that were detectable in both datasets, CNC-exclusive transcripts were enriched in VSMC1 and 2, while SHF-exclusive transcripts were enriched in VSMC3 and 4 (Supplementary Figure 5E). However, the correlation was not perfect, and cluster distribution as defined by scRNA sequencing is likely the result of both embryological origin and anatomical location, suggesting that VSMC heterogeneity in the proximal aorta may be better defined by a gradient of location specific determinants rather than simply by lineage-of-origin.

### Global and Subset-Specific Transcriptional Changes in Response to *Smad3*-Deficiency in VSMCs

**Figure 7:**
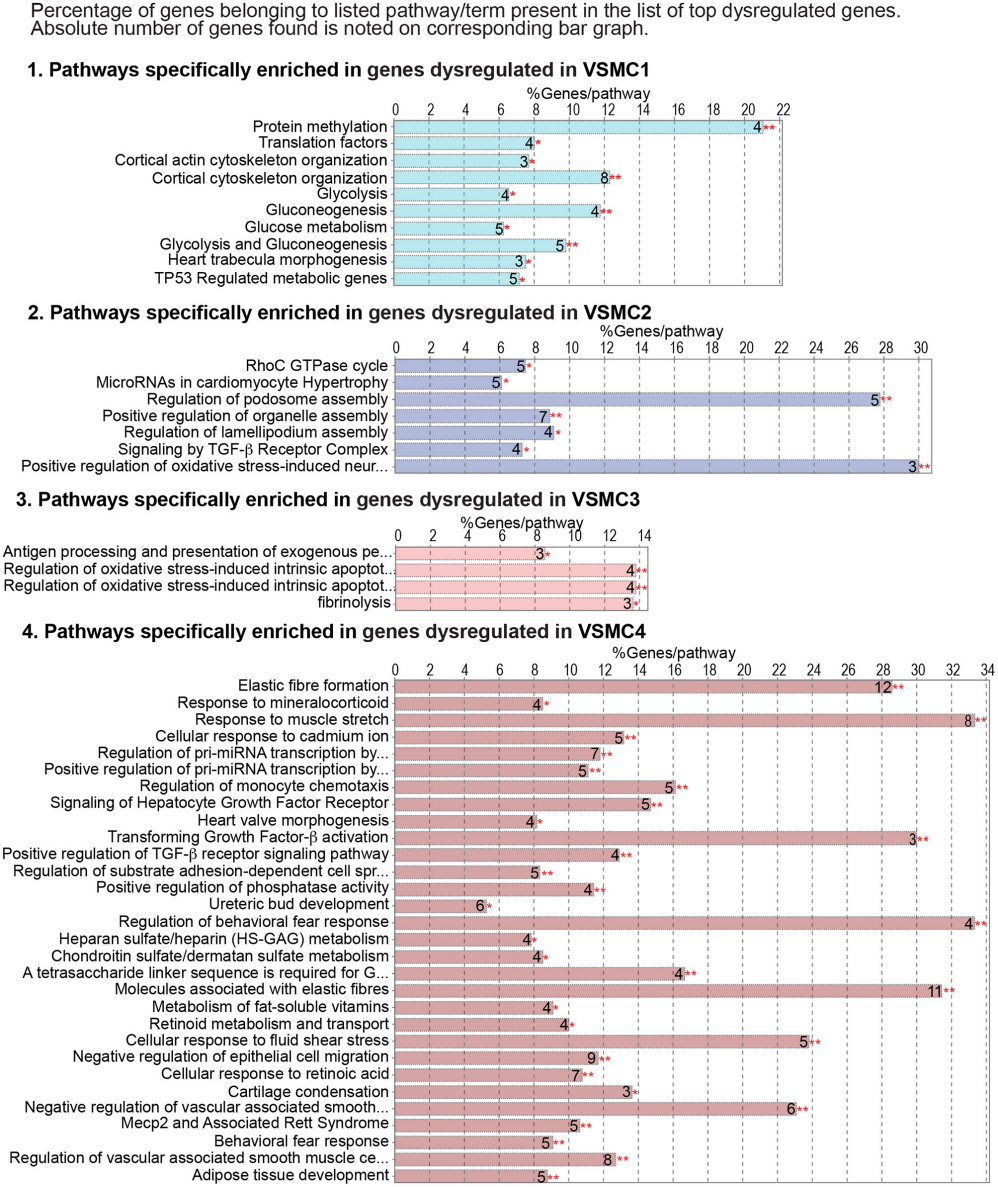
Genes dysregulated in individual VSMC subsets show enrichment for specific pathways. Cluster-specific gene enrichment results as calculated by ClueGO for the top 200 dysregulated transcripts for each VSMC subset. Bar graphs shows the percent of genes belonging to a given term, while the absolute number of genes for each term is indicated as a number next to the corresponding bar. Asterisks refer to the significance of enrichment as calculated by ClueGO algorithm and corrected using the Benjamini-Hochberg method for multiple testing correction.

**Figure 8:**
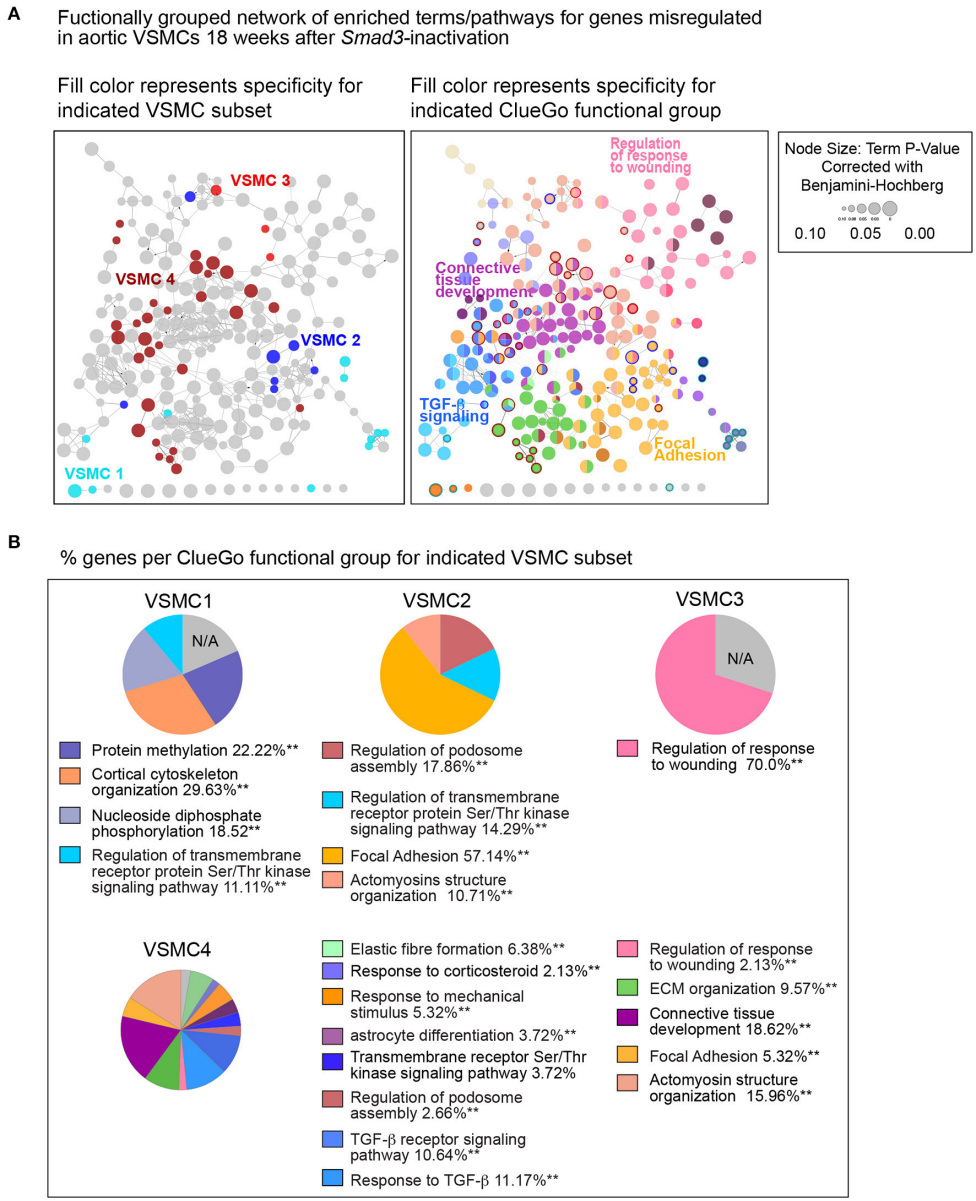
Functional network of global and subset-specific enriched terms for genes dysregulated in response to *Smad3*-deficiency in VSMCs. **(A)** The top 200 dysregulated transcripts for each subset (regardless of direction) were analyzed using the multiple-clusters option in ClueGO, which allows for the simultaneous analysis and visualization of both cluster-specific and non-cluster specific enriched pathways and biological terms in a functional network. In the left panel, enriched nodes are colored based on specificity for a given VSMC subset, while non-specific terms are left grey; on the right panel, network nodes fill color corresponds to the ClueGO functional group to which they belong, with terms belonging to more than one group filled-in by more than one color. Terms and pathways significantly enriched in only one VSMC cluster are identified by the color of the node's border. **(B)** The distribution of genes per ClueGO functional group for each VSMC subset.

To interrogate how intrinsic VSMC heterogeneity contributes to the dysregulation we observed in all *Smad3*-deficient VSMCs at 18 weeks post-deletion, we examined transcriptional changes in the four VSMC clusters, which *in situ* hybridization showed to partly reflect the proximal-to-distal anatomic location. The top 200 dysregulated transcripts for each subset, as defined by absolute Log_2_FC (agnostic as to the direction of change) and *P*-value, were analyzed using the multiple-clusters option in ClueGO, which allows for the simultaneous analysis and visualization of both cluster-specific and non-cluster specific pathways (and/or biological terms) in a functional network (Supplementary Table 7). This analysis revealed the existence of both shared and VSMC-cluster specific enriched pathways (Figures 7, 8), with transcripts misregulated in VSMC2 and VSMC4 showing the highest degree of subset-specific gene enrichment (Figure 7). Functional organization of enriched pathways into ClueGO functional groups showed functional segregation of pathways perturbed in VSMC2 and VSMC4, with perturbation of actomyosin structures and focal adhesion machinery being mostly affected in the former and expression of TGF-β dependent extracellular matrix proteins, including elastic fiber components and collagens, in the latter (Figures 8A,B). VSMC1 also showed perturbation of actomyosin structures, in addition to those related to metabolic regulation. Genes prominently misregulated in VSMC3 were related to response to injury (Figures 8A,B). All clusters except VSMC3 showed dysregulation of genes involved in modulation of signaling by transmembrane receptors with Serine/Threonine kinase activity, which includes signaling by TGF-β Receptors (Figures 8A,B). Taken together, these data indicate that perturbation of TGF-β-signaling by *Smad3* deletion produces both global and subset-specific responses in VSMCs, which may reflect distinct roles played by each subset in the disease process.

### Intrinsic and Disease-Associated Transcriptional Signatures in VSMC Clusters

**Figure 9:**
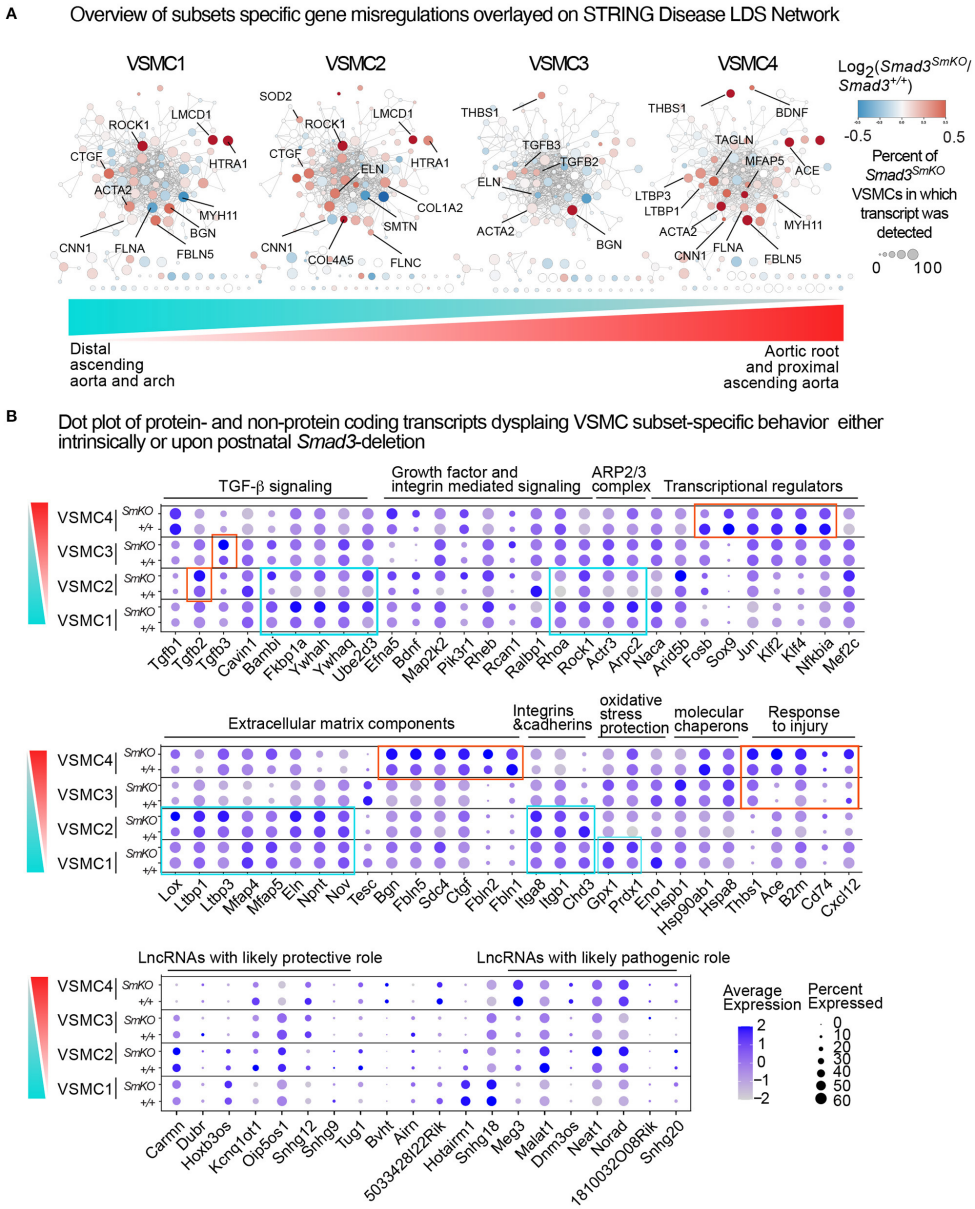
Intrinsic and disease-associated transcriptional signatures in the VSMC subclusters. **(A)** Networks related to LDS1-5 were downloaded from the STRING Disease database, and overlayed with relative expression data for each VSMC subset using Cytoscape. Genes shown in blue are downregulated and genes shown in red are upregulated in *Smad3*^*SmKO*^ relative to controls. Scale indicates the average Log_2_FC in expression in *Smad3*-deficient VSMCs relative to controls for transcripts coding for proteins in the network. Size of the node indicates the percent of cells in the subcluster that express a given transcript. Color bar indicates the gradient of cells populating VSMC subsets in the aortic root and proximal ascending aorta (red) and distal ascending aorta and arch (cyan), with VSMC1 cells being most enriched in the distal ascending aorta and arch and VSMC4 cells being most enriched in the aortic root and proximal ascending aorta. **(B)** Dot plots of protein and non-protein coding transcripts that show VSMC subset-specific behavior. The intensity of the dot's color corresponds to a scaled average expression of each transcript while the size of the dot indicates the percentage of cells in each cluster that express that transcript. Vertical bar indicates the gradient of cells populating VSMC subsets in the aortic root and proximal ascending aorta (red) and distal ascending aorta and arch (cyan).

In order to visualize the behavior of the different VSMC subsets in relation to pathways previously identified as involved in hereditary aneurysm, we downloaded networks related to LDS (LDS1 to LDS5) from the STRING Disease database (37). Expression changes in *Smad3*-deficient VSMCs relative to controls for transcripts coding for proteins in the network were visualized as fill color for each node (Figure 9; Supplementary Figure 6). This analysis revealed striking differences in the behavior of each subset, which included opposite direction of change for transcripts derived from genes critically involved in aneurysmal disease such as *Myh11, Acta2, Flna, Smtn*, and *Tagln* (55–59). For example, *Rock1* was highly upregulated in VSMC1 and 2 but not in VSMC3 and 4. *FlnA*, a target gene of TGF-β/Smad signaling (60) that is necessary for vascular homeostasis (57, 61), was downregulated in VSMC1 and 2 but normalized and upregulated in VSMC3 and 4, respectively. Transcripts coding for *Tgfb2* and *Tgfb3* were upregulated in VSMC3, and those for *Ace* and *Bdnf* in VSMC4. *Thbs1* was notably upregulated in both VSMC3 and VSMC4 (Figure 9A; Supplementary Figure 4), indicating that these subsets are the major contributor of *Thbs1* upregulation observed in the analysis of all VSMCs combined (Figure 5B).

A more detailed analysis of subset-specific changes in our scRNA dataset (Figure 9B) also shows discrete transcriptional patterns both intrinsic and secondary to *Smad3*-deletion in each VSMC cluster. Notable differences include the type of ECM transcript present and/or upregulated in response to *Smad3* deficiency, relative expression of specific integrins, and expression of injury-associated transcripts. Additionally, while VSMC4 cells upregulated putatively pathogenic transcripts associated with vascular injury (*Cd74, Thbs1, Ace, B2m, Cxcl12*) (46, 62–65), VSMC1 upregulated putatively protective genes such as those coding for enzymes that provide protection from oxidative stress (*Gpx1* and *Prdx1*) (66, 67). We also noted that, in response to *Smad3*-deletion, VSMC1 and VSMC2 cells upregulated expression of specific regulators of TGF-β signaling such as *Bambi* (68), *Fkbp1a* (69), *Ywhaq* and *Ywhah* (whose gene product belongs to the 14-3-3 family of multifunctional adaptor proteins) (70), and *Ube2d3* (71). Analysis of long-non-coding RNAs (lncRNAs) detected in our dataset also revealed that differential expression and/or *Smad3*-deficiency induced up- or downregulation in each subset. For example, VSMC4, and to a lesser extent VSMC3, intrinsically expressed lower levels of Cardiac mesoderm enhancer-associated non-coding RNA (*Carmn*), a lncRNA previously shown to have a cardiovascular protective effect (72), and higher expression of maternally expressed 3 (*Meg3*), a lncRNA that regulates VSMC phenotypes and is co-regulator of Smad2/Smad3 transcriptional activity (73, 74).

Although the ultimate combinatorial functional outcome of these subset-specific changes cannot be inferred simply by transcriptional profiling, taken together, these data suggested that the response to the global insult imposed by loss of *Smad3* activity has a heterogenous impact on different VSMC subsets and that subset-specific changes, such as local upregulation of transcripts coding for pro-pathogenic factors such as Thbs1 and ACE (75) as well as intrinsically lower expression of protective factors such as the lncRNA *Carmn* (72), may predispose aortic segments where these cells are most abundant to increased pathogenesis.

## Discussion

Inactivating mutations in genes coding for positive effectors of the TGF-β signaling pathway cause hereditary forms of thoracic aortic disease, including those associated with LDS (2–6, 9, 76–82). The mutational repertoire clearly points to loss of canonical TGF-β signaling as an initiating event in the process that eventually leads to mechanical failure of the aortic wall, dilation, and then aneurysm. However, aneurysmal tissue from both patients and mouse models often shows evidence of either upregulated or normalized TGF-β activity, as measured by levels of Smad2 and Smad3 (Smad2/3) phosphorylation and expression of target genes of the pathway (2–6, 9, 16, 17, 77–80, 83, 84). Although the redundancy and overlapping nature of many signaling pathways in the vascular wall makes it difficult to establish whether upregulation of Smad2/3 phosphorylation and that of target genes (i.e., *Ctgf* , *Serpine1, Col1a1*) truly constitutes a signature of TGF-β signaling upregulation, the phenotype observed in tissue at later stages of disease is functionally indistinguishable from a state of “high TGF-β signaling.” Although this secondary compensatory response and associated features are broadly recognized, debate exists as to whether it represents a mostly adaptive, maladaptive, or simply irrelevant feature of aneurysm progression (85, 86).

Understanding the mechanisms and consequences of this transition is complicated by the fact that in mouse models carrying germline aneurysm-causing mutations, the transition from the initial deficiency in TGF-β/Smad signaling to moderate disease and eventual overt disease is entwined with prenatal and perinatal developmental processes that regulate morphogenesis and postnatal remodeling of the aortic wall (87–89). While other laboratories have shown that full postnatal ablation of TGF-β signaling by deletion of the gene coding for TGF-β Receptor II (12, 13, 90) causes quick mechanical failure of the aortic wall, such a severe provocation does not recapitulate the process of disease seen in patients carrying mutations that only partly impair TGF-β signaling output. In the context of LDS-causing mutations, retention of partly and/or fully functional signaling branches leaves open the possibility of secondary signaling upregulation that is not possible when TGF-β Receptor II signaling capability is fully inactivated. Of note, the work of Cook and colleagues in a mouse model of Marfan syndrome strongly indicated that whereas TGF-β signaling had a protective role at early stages of disease, its activation contributed to disease progression at later stages (91), suggesting that a similar process may also apply to aortic pathogenesis in LDS.

On the basis of these considerations, we set out to assess whether partial inhibition of the pathway by postnatal *Smad3* inactivation (with TGF-β Receptor still able to activate Smad2) was sufficient to cause disease, and, if so, whether disease progression was also accompanied by a transition to an apparent excessive signaling, presumably mediated by Smad2. *Smad3* inactivation at 6-weeks of age led to detectable aortic dilation within approximately 18 weeks, and to apparent defects in the aortic wall ultrastructure within 10 weeks (Figures 1, 2, 4). Although hereditary aneurysm in humans is caused by heterozygous, rather than bi-allelic, loss-of-function mutations in *SMAD3* (4), neither tamoxifen-treated *Smad3*^+/*flox*^ *Myh11Cre*^*ER*^ nor mice heterozygous for a null *Smad3* allele (not shown) developed dilation, suggesting a greater compensatory role for murine *Smad2*.

*Smad3*-inactivation causes broad reduction in expression of ECM matrix proteins, including those necessary for assembly and maturation of elastic fibers, as well as that of integrins, focal adhesion adaptor proteins, and selected cytoskeletal proteins (Figure 3; Supplementary Figure 4), all of which was reflected histologically by loss of connections between VSMCs and the elastic lamellae (Figure 4). These observations are consistent with a model in which TGF-β signaling is necessary for expression of genes that modulate assembly and maturation of focal adhesions between VSMCs and elastic lamellae (15, 92–94). This would include genes coding for contractile proteins such as smooth muscle actin (*Acta2*, also known as α-SMA) and smooth muscle myosin heavy chain (*Myh11*, also known as SM-MHC), given that myosin-dependent force generation regulates focal adhesion function by provoking tension-dependent conformational alterations in both integrins and associated signaling anchoring proteins (48, 95–98). Although our data is consistent with a model in which TGF-β signaling deficiency leads to dilation by inducing loss of the elastin-contractile unit (92), evidence from mouse models that are deficient in elastin (*Eln*), the principal component of elastic fibers, indicates that mere loss of connections between VSMCs and elastic lamellae is insufficient to cause aortic dilation. VSMC-specific elastin deficiency results in absence of elastic lamellae and thickening of the ascending aorta but not dilation or aneurysm (99). Additionally, in humans, elastin insufficiency is associated with inherited obstructive arterial disease but not aneurysm (100–107). The subsets of mutations in the elastin *ELN* gene that are known to cause aortic dilatation and rupture in patients are frameshift mutations predicted to result in abnormal tropoelastin monomer maturation into mature elastin, and thus deposition of abnormal elastin that may trigger cellular responses not caused by simple deficiency (107).

Thus, we favor a model whereby loss of proper contacts between VSMCs and the ECM is necessary but not sufficient to cause aortic dilation, with maladaptive compensatory responses to this initial loss being critical to disease progression. The broad “reversal” in expression of many of the same ECM actomyosin, and focal adhesion components, diminished at the 10-week time point and normalized and upregulated at the 18-week time point (Figures 3, 5), corresponds to the onset of detectable dilation in our mouse model, and seems to indicate that this attempt at compensation is part of the disease process. We propose that abnormal signaling downstream of integrins and focal adhesions, triggered by an irreparably dysfunctional matrix, and possibly imbalanced intracellular repertoire of signaling and adaptor proteins, is a key driver of disease progression. The reversal in expression of many components and regulators of these complexes was also accompanied by increased expression of TGF-β ligands as well as that of activators of the angiotensin II signaling pathway, such as transcripts coding for ACE and Thbs-1, all of which can promote pathogenic remodeling of the extracellular matrix (46, 108–110). Unfortunately, levels of *Agtr1a*, which codes for the angiotensin II Type I Receptor, a modulator of several pathogenic pathways driving aneurysm progression, were too low to be detected in this single cell transcriptomic analysis (86).

We also examined how VSMC heterogeneity may explain the peculiar vulnerability of the aortic root to dilation, observed both in our mouse model as well as in patients carrying analogous mutations (8). The proximal thoracic aorta is populated by VSMCs derived from cardiac neural crest (CNC) (111) and second heart field (SHF)-derived progenitors (54, 112). Our own work and that of others has shown that these two types of VSMCs respond differently to aneurysm-causing mutations, including those that cause Smad3 deficiency, and suggested the possibility that their peculiar distribution in the aortic root and ascending aorta may underlie regional susceptibility to aneurysmal disease (11, 84, 110, 113–115) (Supplementary Figure 5).

In our analysis, the VSMC clusters identified by scRNA sequencing analysis correlated but did not overlap with embryological lineage (Supplementary Figure 5). Cells expressing cluster-defining transcripts for each of the four VSMC clusters were present within the aortic media and distributed in a gradient along the distal to proximal axis, with cells expressing cluster-defining transcripts for VSMC3 and 4 being most abundant, but not exclusive, to the aortic root, and those expressing cluster-defining transcripts for VSMC1 and 2 being more abundant in the proximal and distal ascending aorta (Figure 6). We interpret the four VSMC clusters as being a function of both intrinsic factors derived from lineage-of-origin and extrinsic factors due to anatomical location, with influences of both blood flow and other environmental factors such as longitudinal and circumferential stretch imposed by heart contraction (116, 117). Although existence of a population of modulated VSMCs has been reported in the context of aneurysm in a mouse model of Marfan Syndrome (110), we did not find this population in our analysis, possibly because aortic dilation, even at 18 weeks, is still too mild in our model and the percent of modulated VSMCs, which has been described to increase with time, is still too low.

**Figure 10:**
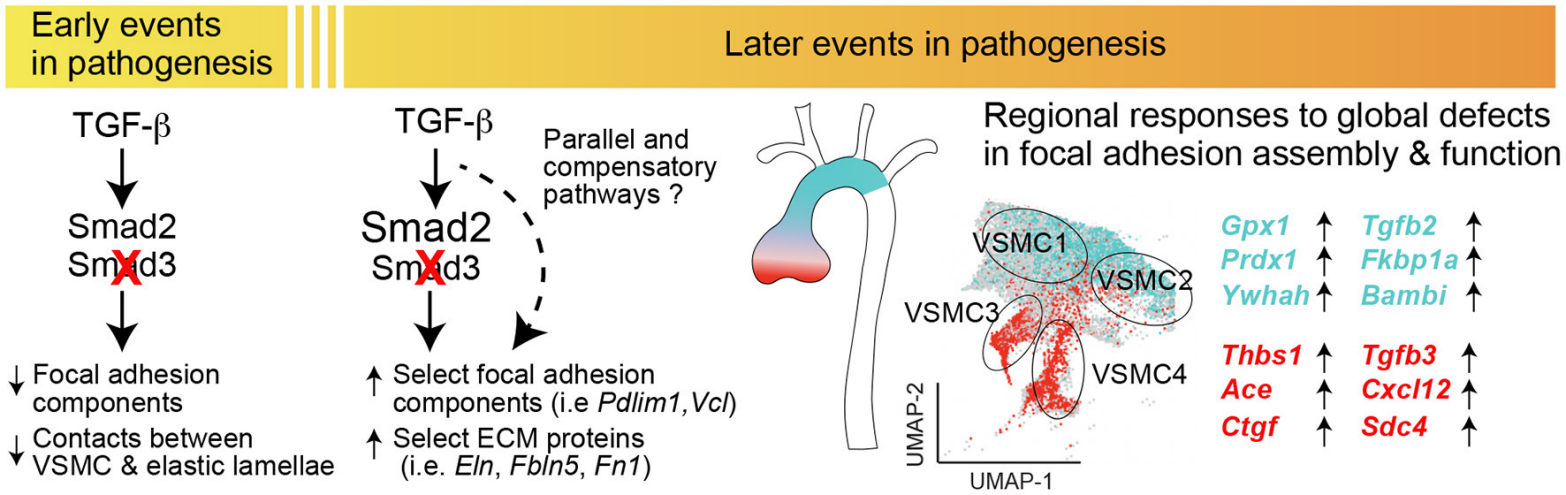
Postnatal *Smad3* inactivation in smooth muscle cells elicits a temporally and regionally distinct pathogenic response. Diagram summarizing the major findings of the study. *Smad3* inactivation in VSMCs results in a broad reduction in the expression of components of the extracellular matrix and focal adhesions, including integrins and anchoring proteins, and results in loss of connections between VSMCs and elastic lamellae. Later in the disease process, normalization or upregulation of transcripts belonging to the same functional categories associates with upregulation of transcripts coding for putatively pro-pathogenic factors such as *Ace, Thbs1, Cxcl12* as well as that of TGF-β ligands; these changes are most pronounced in VSMC subsets enriched in the aortic root, while upregulation of putatively protective transcripts is more pronounced in VSMC subsets enriched in the ascending aorta, which is less prone to dilation in this model. These data suggest that loss of functional matrix and focal adhesions has secondary consequences specific to each VSMC subset, possibly contributing to the regional susceptibility to dilation in the aorta.

Analysis of transcripts misregulated after *Smad3* deletion shows both shared and VSMC subset-specific alterations, including transcripts coding for a network of proteins previously shown to be involved in disease progression in LDS (STRING Disease LDS Network, Figure 9; Supplementary Figure 6). This subset-specific behavior may explain discordant findings among laboratories with respect to expression of some of these disease markers, given that assessment of expression, and possibly functional outcome, would be dependent on the precise mix of VSMCs that are assessed. The precise effect that these subset-specific differences have on the disease process are hard to predict simply on the basis of the transcriptional analysis described in this study; it is notable, however, that VSMC subsets more abundant in relatively protected portions of the aorta also express increased levels of likely protective factors, and show a distinct expression pattern for both matrix proteins and modulators of focal adhesions (Figures 7–9), while the converse is true for VSMC subsets more abundant in the aortic root, where disease is more pronounced (Figure 10).

The fact that these four VSMC subsets exist in control animals suggests that distinct properties of the aortic root as determined by the proportion of specific VSMC subsets, specifically VSMC4, VSMC3 and VSMC2, distinguishes this segment prior to any disease-causing insult, and renders it intrinsically more vulnerable to the effect of *Smad3* inactivation. We speculate that different VSMC subsets may confer specific susceptibility to genetic and/or environmental factors that preferentially affect a given aortic segment, and that a better understanding of the molecular determinants of these vulnerabilities may guide development of specific therapies that target maladaptive responses that contribute to disease progression.

## Data Availability Statement

The datasets presented in this study can be found in NCBI's Gene Expression Omnibus (GEO) database under the following accession numbers: GSE189025 and GSE189544.

## Ethics Statement

The animal study was reviewed and approved by Animal Care and Use Committee at Johns Hopkins University School of Medicine.

## Author Contributions

EB and TC have contributed equally to this work and share first authorship. EM is responsible for the conception and design of this study, prepared slides, and acquired microscope images. TC initiated the experimental work, including animal breeding, performed echocardiography for most of the animals and produced the initial single cell RNA (scRNA) sequencing dataset and preliminary analysis. EB assisted with echocardiography and animal breeding and contributed significantly to the analysis of the scRNA sequencing data. WC contributed to the analysis of the scRNA sequencing data and performed the *in situ* validation. LG provided guidance in the processing and analysis of the scRNA sequencing data. MS assisted in all phases of this work including animal breeding and genotyping, performed the histological staining, and collected the blood pressure measurements. EB assisted EM in creating figures and wrote the majority of the manuscript. WC, RB, TC, and MS wrote portions of the methods. RB performed the bulk RNA sequencing which was analyzed by EM and TC. LR stained and imaged aortic sections for electron microscopy and quantified the number of connections in the resulting electron micrographs. All authors contributed to manuscript revision, read, and approved the submitted version.

## Funding

Research reported in this publication was supported by the National Heart, Lung, and Blood Institute of the National Institutes of Health under Award Number R01HL147947 and by a generous gift from the Loeys-Dietz Foundation. EM was also supported by funding provided to Johns Hopkins by the Broccoli family.

## Conflict of Interest

The authors declare that the research was conducted in the absence of any commercial or financial relationships that could be construed as a potential conflict of interest.

## Publisher's Note

All claims expressed in this article are solely those of the authors and do not necessarily represent those of their affiliated organizations, or those of the publisher, the editors and the reviewers. Any product that may be evaluated in this article, or claim that may be made by its manufacturer, is not guaranteed or endorsed by the publisher.
