# Supplementary material for: Postnatal Smad3 Inactivation in Murine Smooth Muscle Cells Elicits a Temporally and Regionally Distinct Transcriptional Response
Source: Front Cardiovasc Med. 2022 Apr 8;9:826495. doi: 10.3389/fcvm.2022.826495 (PMC9033237; doi:10.3389/fcvm.2022.826495)
Supplement: Supplementary file 8 [file Data_Sheet_1.PDF]

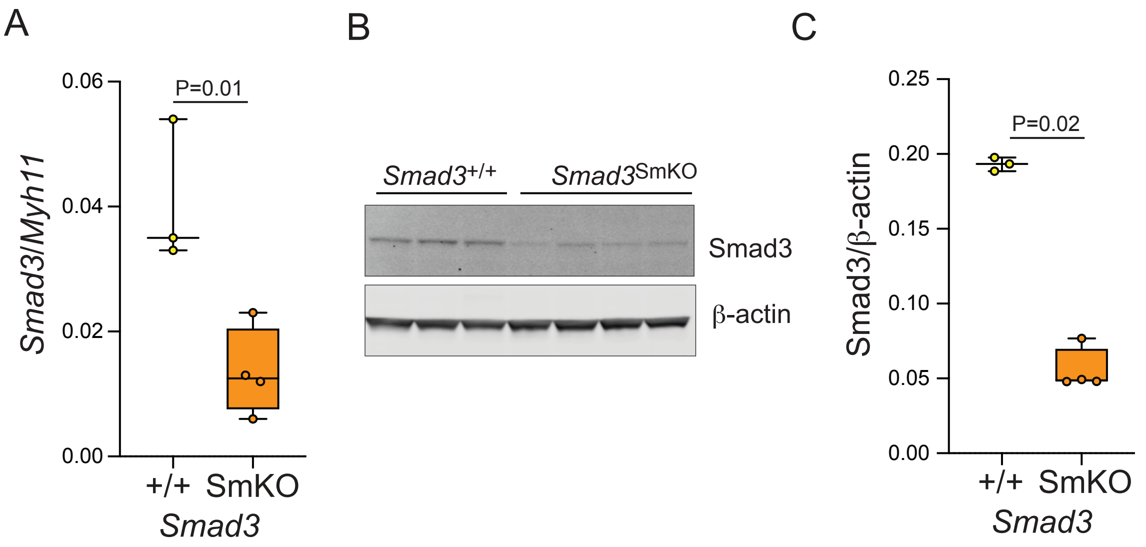

Supplemental Figure 1. Validation of *Smad3* inactivation in *Smad3*^{SmKO} mice by qPCR and immunoblot. (A) qPCR analysis of *Smad3* relative to *Myh11* expression in the aortas of *Smad3*$^{+/+}$ (n=3) and *Smad3*^{SmKO} (n=4) mice. **(B)** Immunoblot of Smad3 expression relative to β-actin in aortic lysates of *Smad3*$^{+/+}$ (n=3) and *Smad3*^{SmKO} (n=4) mice. (**C**) Quantification of immunoblot shown in B. P-values refer to two tailed Student's t-test.
