# Supplementary material for: Postnatal Smad3 Inactivation in Murine Smooth Muscle Cells Elicits a Temporally and Regionally Distinct Transcriptional Response
Source: Front Cardiovasc Med. 2022 Apr 8;9:826495. doi: 10.3389/fcvm.2022.826495 (PMC9033237; doi:10.3389/fcvm.2022.826495)
Supplement: Supplementary file 9 [file Data_Sheet_2.PDF]

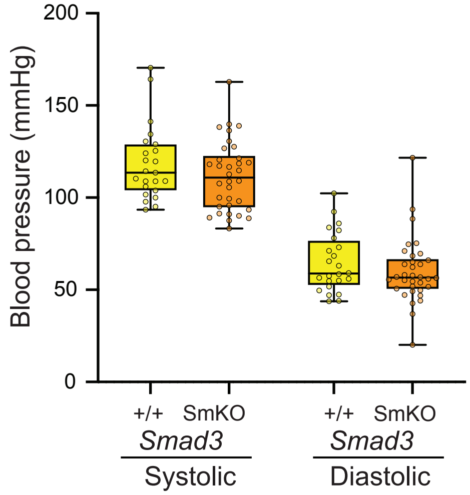

Supplemental Figure 2. Postnatal *Smad3* deletion does not alter blood pressure.
Systolic and diastolic blood pressure of *Smad3*$^{+/+}$ and *Smad3*^{SmKO} mice measured at 24 weeks of age.
