# Supplementary material for: Postnatal Smad3 Inactivation in Murine Smooth Muscle Cells Elicits a Temporally and Regionally Distinct Transcriptional Response
Source: Front Cardiovasc Med. 2022 Apr 8;9:826495. doi: 10.3389/fcvm.2022.826495 (PMC9033237; doi:10.3389/fcvm.2022.826495)
Supplement: Supplementary file 10 [file Data_Sheet_3.PDF]

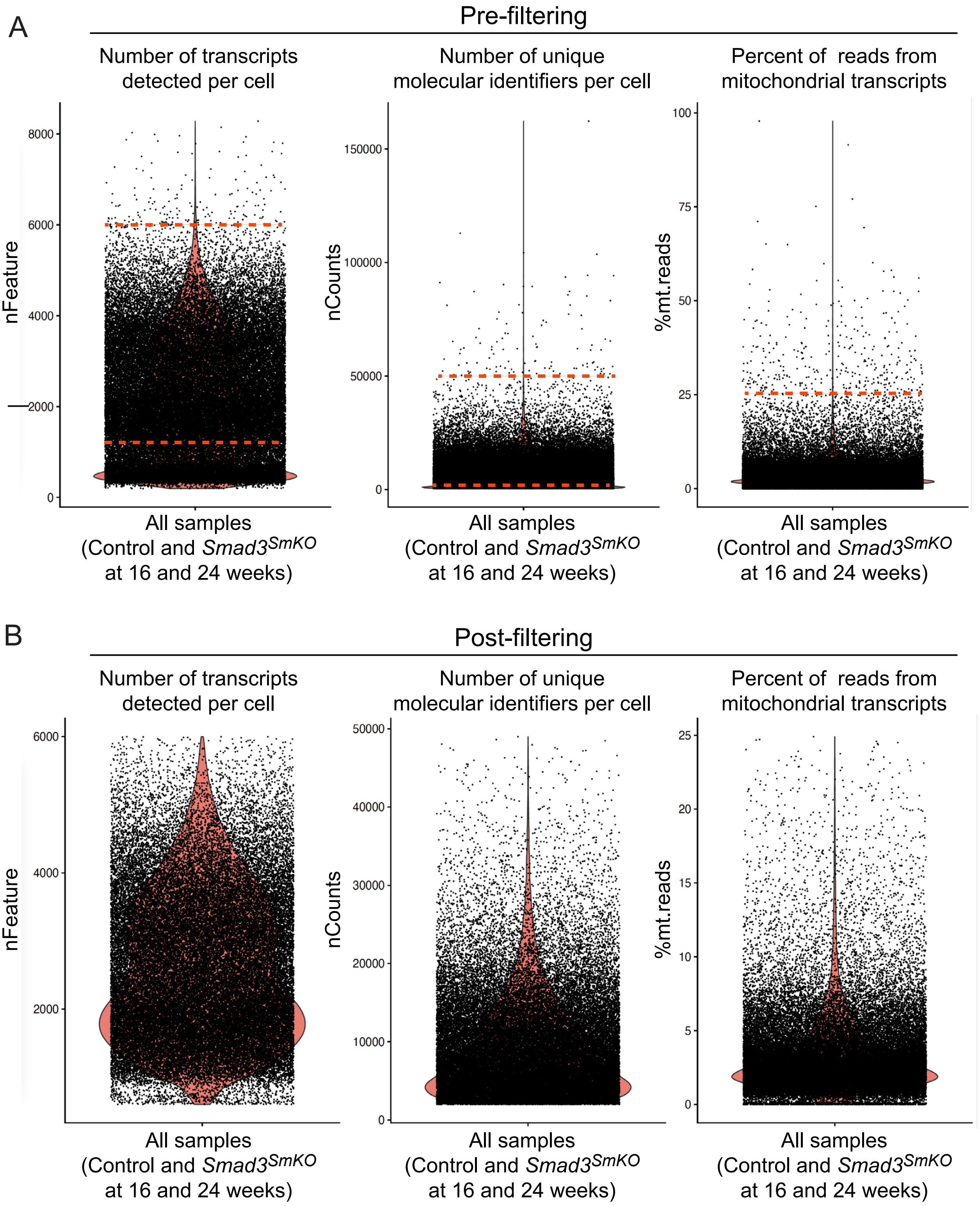


**Supplemental Figure 3. Quality control and filtering of single cell RNA sequencing data by number of transcripts per cell, number of molecules per cell, and mitochondrial ratio. (A)** Density plots indicating the distribution of cells with respect to the number of transcripts detected per cell (nFeature), the number of unique molecular identifiers per cell (nCounts), and the percentage of mitochondrial transcripts detected per cell (%mt.reads) of the entire single cell RNA sequencing dataset prior to filtering, which includes 66,261 live, nucleated aortic cells from 16 mice. To eliminate cells that may be dying or that resulted from an empty droplet those with an nFeature below 600 and/or an nCounts below 2000 were removed. To eliminate cells that may be doublets or multiplets (more than one cell partitioned into a single droplet) those with an nFeature greater than 6000 and/or an nCounts greater than 50000 were removed. Cells with greater than 25% of their reads mapping to mitochondrial transcripts, indicating poor cell quality, were removed. 50,835 cells were retained for subsequent analysis. **(B)** Density plots for nFeature, nCounts, and %mt.reads post-filtering.
