# Supplementary material for: Postnatal Smad3 Inactivation in Murine Smooth Muscle Cells Elicits a Temporally and Regionally Distinct Transcriptional Response
Source: Front Cardiovasc Med. 2022 Apr 8;9:826495. doi: 10.3389/fcvm.2022.826495 (PMC9033237; doi:10.3389/fcvm.2022.826495)
Supplement: Supplementary file 11 [file Data_Sheet_4.PDF]

Supplemental Figure 4

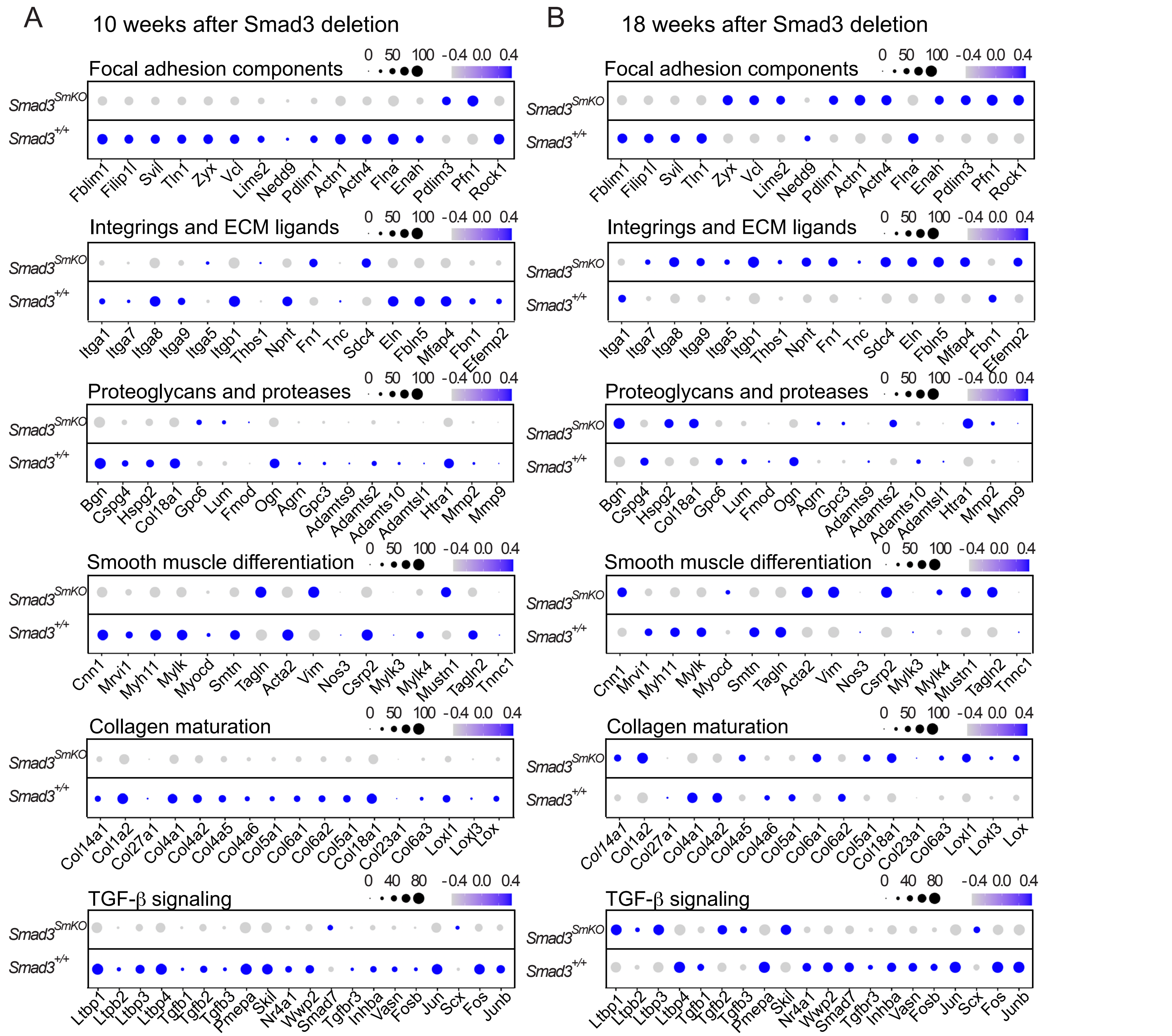

Supplemental Figure 4. Early global defects in focal adhesion machinery and ECM components drive time-sensitive adaptive and maladaptive transcriptional upregulation in response to *Smad3*-deficiency in VSMCs. Dot plots showing expression of relevant transcripts in *Smad3*$^{+/+}$ and *Smad3*^{SmKO} VSMCs at **(A)** 10- and **(B)** 18-weeks post-deletion.
