# Supplementary material for: Postnatal Smad3 Inactivation in Murine Smooth Muscle Cells Elicits a Temporally and Regionally Distinct Transcriptional Response
Source: Front Cardiovasc Med. 2022 Apr 8;9:826495. doi: 10.3389/fcvm.2022.826495 (PMC9033237; doi:10.3389/fcvm.2022.826495)
Supplement: Supplementary file 12 [file Data_Sheet_5.PDF]

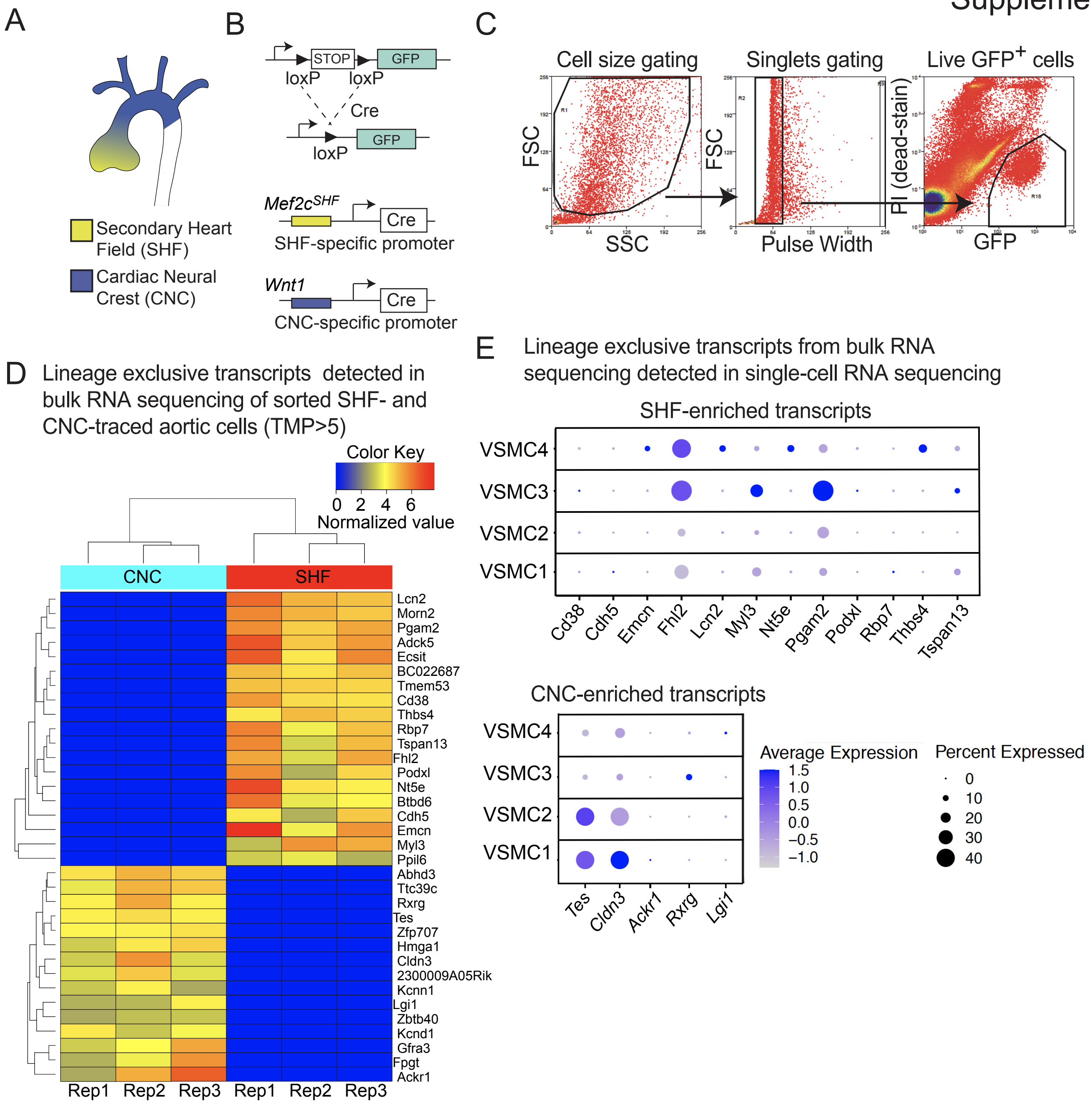

Supplemental Figure 5. Bulk RNA sequencing of SHF and CNC traced VSMCs reveals lineage-exclusive transcripts that are enriched in specific VSMC subsets.
(A) Schematic showing the approximate distribution of Secondary Heart Field (SHF)-derived and Cardiac Neural Crest (CNC)-derived vascular smooth muscle cells (VSMCs) in the aorta. **(B)** Construct of a Cre-mediated recombination induced GFP (green fluorescent protein) reporter used with *Mef2c-Cre* and *Wnt1-Cre* to trace SHF and CNC cells, respectively. **(C)** Flow cytometry gating used to sort populations of living SHF and CNC-traced cells for bulk RNA sequencing. FSC- forward scatter, SSC- side scatter. **(D)** Heat map showing SHF and CNC-exclusive transcripts in bulk RNA sequencing analysis of SHF and CNC-traced VSMCs. Three biological replicates were included per group. **(E)** Dot plot showing expression of SHF and CNC-exclusive transcripts that were detected in the single cell transcriptomic analysis across all VSMC subclusters. The intensity of the dot's color corresponds to a scaled average expression of each transcript while the size of the dot indicates the percentage of cells in each cluster that express that transcript.
