# Supplementary material for: Postnatal Smad3 Inactivation in Murine Smooth Muscle Cells Elicits a Temporally and Regionally Distinct Transcriptional Response
Source: Front Cardiovasc Med. 2022 Apr 8;9:826495. doi: 10.3389/fcvm.2022.826495 (PMC9033237; doi:10.3389/fcvm.2022.826495)
Supplement: Supplementary file 13 [file Data_Sheet_6.PDF]

Supplemental Figure 6

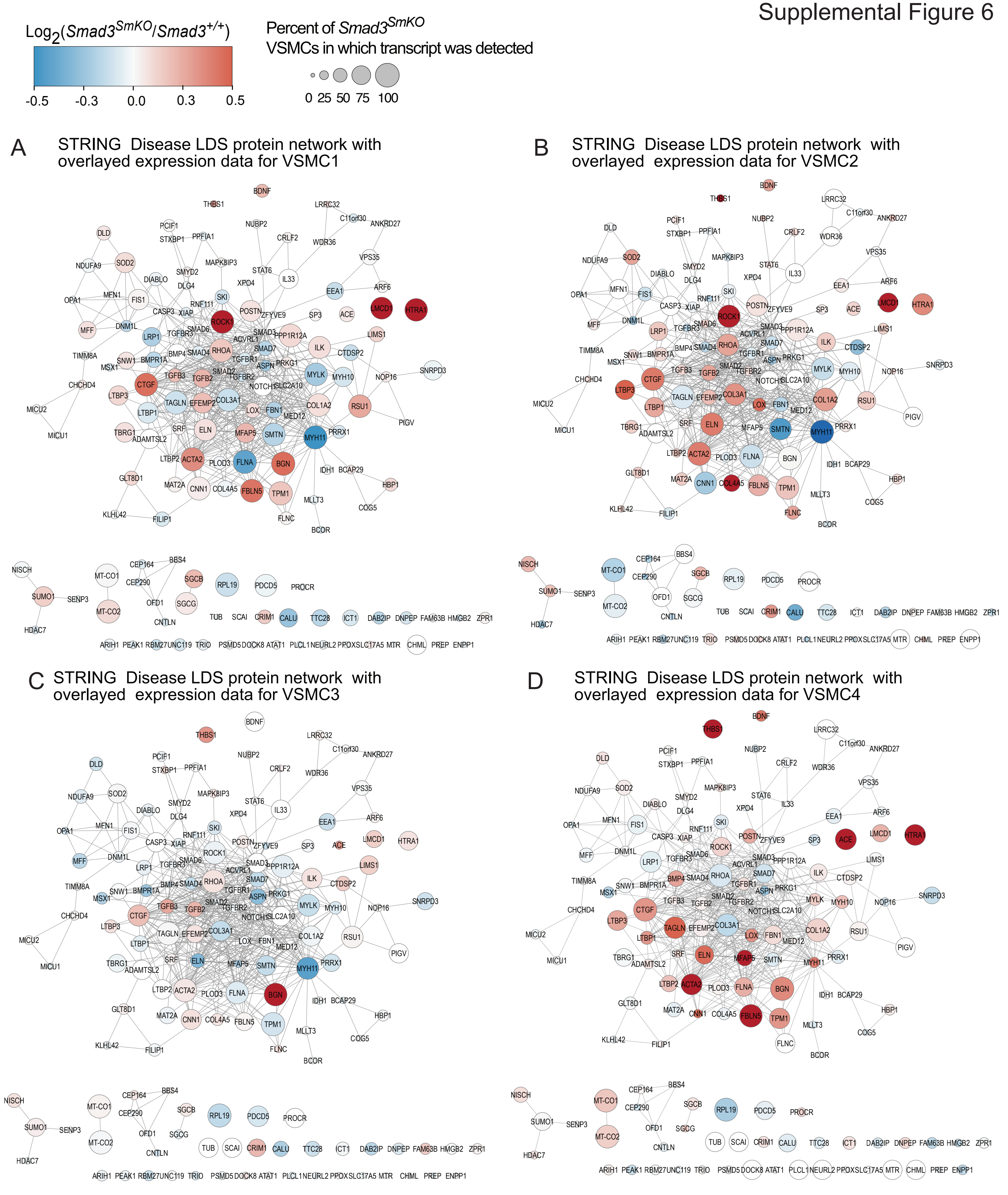

Supplemental Figure 6. VSMC subset-specific expression of transcripts associated with LDS in the STRING Disease database. Networks related to LDS1-5 were downloaded from the STRING Disease database and the full network was overlayed with relative expression data for **(A)** VSMC1, **(B)** VSMC2, **(C)** VSMC3, and **(D)**VSMC4 using Cytoscape. Genes shown in blue are downregulated and genes shown in red are upregulated in *Smad3*^{SmKO} relative to controls. Scale indicates the average Log_2FC in expression in *Smad3*-deficient VSMCs relative to controls for transcripts coding for proteins in the network. Size of the node indicates the percent of cells in a the subcluster that express a given transcript.
